# Unravelling friction anisotropy by atomic force microscopy

**DOI:** 10.1111/jmi.70073

**Published:** 2026-03-28

**Authors:** Clodomiro Cafolla, Marcello Campione

**Affiliations:** ^1^ Durham Physics Department Durham England; ^2^ Dipartimento di Fisica ‘E. Fermi’ Università di Pisa Pisa Italy; ^3^ Department of Earth and Environmental Sciences University of Milano‐Bicocca Milano Italy

**Keywords:** confined liquids, crystalline surfaces, friction anisotropy and asymmetry, lubrication, 2D materials, nanoscale tribology, polymers, scanning probe microscopy, van der Waals materials

## Abstract

Friction plays a crucial role in both natural phenomena, from the flow of blood cells to earthquakes, and technological applications, from car engines to wind turbines. One of the most fundamental aspects of tribology is friction anisotropy, that is, the dependence of the friction force vector on the direction of sliding. Even when two identical surfaces slide against each other, differences in sliding direction can lead to significantly different friction forces.

While atomistic and semi‐empirical models provide a good understanding of friction and friction anisotropy at the molecular and macroscale levels, a comprehensive understanding at the nano‐ and microscale remains elusive. Unravelling the mechanisms of friction anisotropy at these intermediate length scales is crucial for bridging the gap between the current atomistic and macroscale models, as well as for advancing technological applications, such as nano‐/micro‐electromechanical systems (NEMS/MEMS).

One experimental technique which has significantly contributed to the study of nano‐ and micro‐scale friction anisotropy is scanning probe microscopy, particularly the form of atomic force microscopy (AFM). AFM enables investigations at the nanometre‐scale resolution, providing at equilibrium topographical, physical, and chemical information of interfaces with atomic precision. AFM can also measure friction forces with pN‐level sensitivity while operating in vacuum, air or fluid environments. The versatility of AFM and its applicability to both soft and hard materials have made it an indispensable tool for understanding the nano‐ and microscale mechanisms of friction.

In this review, we examine the contributions of AFM‐based techniques to the study of friction anisotropy. First, we summarise key AFM findings on friction anisotropy arising from the periodic corrugation of atomically flat crystals, quasicrystals, 2D materials and organic materials. In the second part, we explore friction anisotropy mechanisms influenced by the presence of solid or fluid adsorbates, such as polymers or liquid lubricants.

By synthesising insights from the last 30 years of research, our review contributes to a deeper understanding of friction anisotropy, identifying common mechanisms across the diverse systems. This knowledge will not only refine theoretical models but also drive technological advancements, in a wide range of applications from biomedical devices and 3D printing to engines and advanced lubricant formulations.

## 1 INTRODUCTION

Friction is ubiquitous in nature and technology,^1^, ^2^ influencing a vast range of phenomena and devices, from cell rolling^3^ and nano/micro‐electromechanical systems (NEMS/MEMS),^4^ to car and machinery engines.^4^, ^5^ Its significance is highlighted by the fact that it accounts for over 20% of global energy waste annually.^4^, ^5^ Understanding the underlying mechanisms of friction is therefore crucial for optimising lubricant design and developing efficient tribological solutions.^4^, ^5^, ^6^, ^7^, ^8^, ^9^, ^10^, ^11^, ^12^

In this context, scanning probe microscopy (SPM) techniques, particularly atomic force microscopy (AFM), provide an invaluable tool for investigating friction at the nano‐ and microscale. AFM enables atomic‐resolution imaging of contact surfaces,^4^, ^13^, ^14^, ^15^, ^16^, ^17^, ^18^, ^19^, ^20^, ^21^, ^22^, ^23^, ^24^, ^25^ while measuring friction forces with pN‐level precision.^26^, ^27^, ^28^, ^29^, ^30^, ^31^, ^32^, ^33^, ^34^, ^35^, ^36^, ^37^, ^38^, ^39^, ^40^ AFM furthermore allows direct visualisation of fluid lubricant organisation at the solid/liquid interface, and precise quantification of their dynamic response under an applied load or shear.^17^, ^18^, ^22^, ^23^, ^26^, ^34^, ^41^

Since its invention in 1986,^42^ AFM has indeed played a transformative role in uncovering molecular and nanoscale friction mechanisms.^4^, ^10^, ^26^, ^27^, ^32^, ^40^, ^43^, ^44^, ^45^, ^46^, ^47^, ^48^, ^49^, ^50^, ^51^, ^52^, ^53^, ^54^, ^55^, ^56^ One particularly striking contribution is its ability to study friction anisotropy – the dependance of the friction force vector on the direction of sliding.^4^, ^10^, ^27^, ^57^, ^58^, ^59^, ^60^, ^61^, ^62^, ^63^, ^64^, ^65^, ^66^, ^67^, ^68^ Closely related to the concept of friction anisotropy, friction asymmetry refers to the difference in lateral force experienced when scanning the surface in the forward versus the backward direction.^60^, ^69^

**FIGURE 1:**
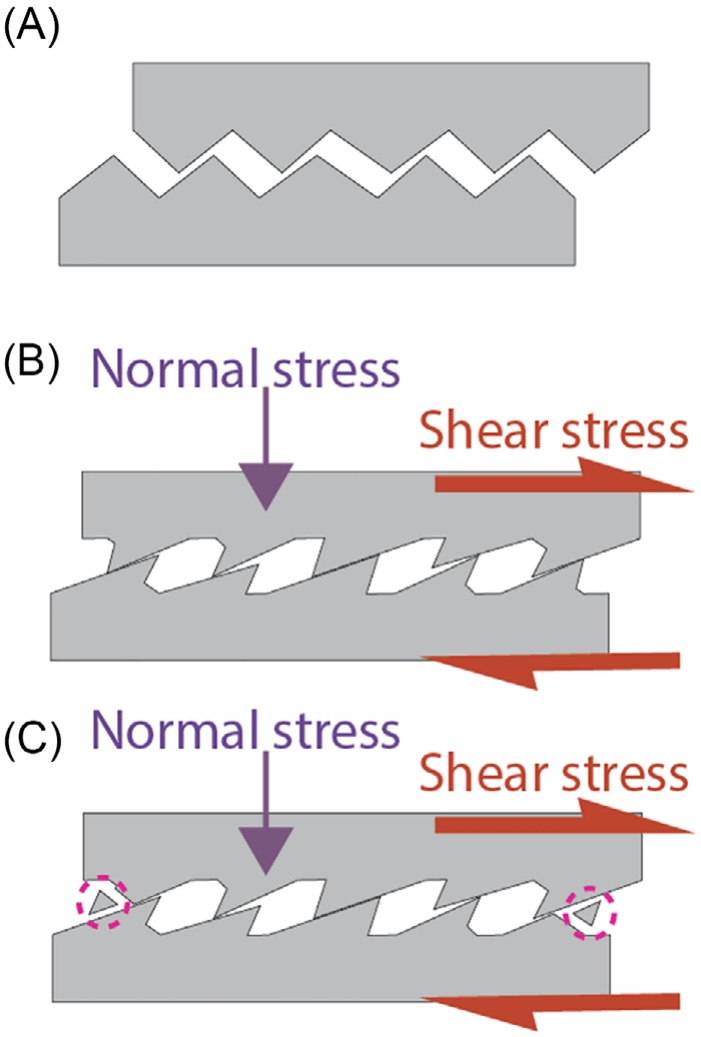
Macroscale friction involves several key factors: (A) the friction force arises from the shear stress required to lift the sliding objects over surface irregularities. (B) For plastic surfaces, this energy cost is primarily due to their deformation. (C) A further contribution to friction results from the breaking of asperities as indicated by the dashed purple circles.

At the macroscopic level, friction anisotropy and asymmetry arise from surface topographical inhomogeneities: the topography of two sliding solids influences friction forces based on the direction of motion, with an energy cost associated with plastic deformation, asperity breakage or both^4^, ^70^, ^71^ (Figure 1). Friction indeed arises from interactions between two surfaces at their contacting asperities. When a tangential force is applied to slide one object over the other, shear stresses develop at the junction interfaces to resist this force (Figure 1B). The resulting friction force is determined by the product of the shear stress required to initiate and sustain sliding and the area over which it acts. For a constant nominal area, an increase in normal stress leads to a greater number of contacting asperities causing friction force to scale proportionally with load while remaining independent of area.^1^, ^4^, ^9^ An additional source of dissipation and friction arises from plastic deformation during sliding (Figure 1C).

Simply scaling down the macroscopic principles that govern friction is insufficient to fully understand its behaviour at the atomic scale.^1^, ^9^, ^72^, ^73^, ^74^ Here, friction anisotropy is primarily governed by atomic arrangement and, in the case of crystalline materials, by the commensurability between the lattice periodicities of the two contacting surfaces.^4^, ^9^ When two atomically flat surfaces come into contact with each other, the atoms of one surface settle into the interatomic gaps of the other, creating an interlocking potential that depends on their relative atomic alignment.^9^, ^74^ Similar mechanisms have been proposed to explain friction asymmetry, attributing the difference in potential experienced by the scanning probe during forward and backward motion to the atomic arrangement of the surface and its interaction with the AFM tip.^75^, ^76^, ^77^ Several theoretical models, including the Prandtl–Tomlinson and the Frenkel–Kontorova frameworks,^1^, ^4^, ^9^, ^78^ have been developed to describe atomic‐scale friction anisotropy and asymmetry. Current models however struggle to fully capture realistic three‐dimensional scenarios,^4^, ^9^ particularly in the presence of fluid lubricants between two sliding solids,^27^ adding further complexity to the problem.

AFM has played a key role in bridging the gap between atomistic models of friction anisotropy/asymmetry and macroscale observations. It has provided insights into how material properties and interface singularities^79^, ^80^ influence friction, while also clarifying general mechanisms such as lubricant molecular ordering^27^, ^61^, ^62^ and variations in the potential energy landscape of contacting surfaces.^50^, ^76^, ^81^

In this review, we explore SPM studies, particularly those employing AFM, that have advanced our understanding of friction anisotropy and asymmetry over the past 30 years, with a primary focus on friction anisotropy. The review is divided into two main sections: (1) friction anisotropy and asymmetry arising from periodic corrugation of atomically flat crystals, quasicrystals, 2D materials and organic materials; (2) friction anisotropy/asymmetry mechanisms influenced by solid or fluid adsorbates, for example, polymers and liquid lubricants.

By consolidating key findings, this review aims to further fundamental insights into nanoscale friction and lubrication while also supporting the design of advanced technological applications. These include catalysis,^82^ solar cells,^83^ organic electroluminescence,^82^, ^84^  NEMS/MEMS,^4^, ^85^ biomedical devices,^86^, ^87^, ^88^ and 3D printing.^4^

## 2 PERIODIC CORRUGATION AND SURFACE SINGULARITIES

### 2.1 Atomic crystals and quasicrystals

One of AFM's most significant contributions to the study of friction anisotropy is its experimental confirmation of theoretical predictions^4^, ^74^, ^89^ regarding the role of periodic corrugation and atomic structure in modulating friction magnitude and anisotropy on atomically flat surfaces.^90^ Commensurability, that is, a perfect match between the lattice points of the two contacting surfaces, results in strong interlocking and relatively high friction during sliding.^4^, ^68^, ^91^ In contrast, for incommensurate surface lattices, sliding occurs with relatively lower friction.^4^, ^92^ In the limiting cases of incommensurability and adiabatic atomic motion, a near‐frictionless friction state known as superlubricity can be achieved,^93^ characterised by a coefficient of friction (COF) < 10^−3^.^4^, ^94^

**FIGURE 2:**
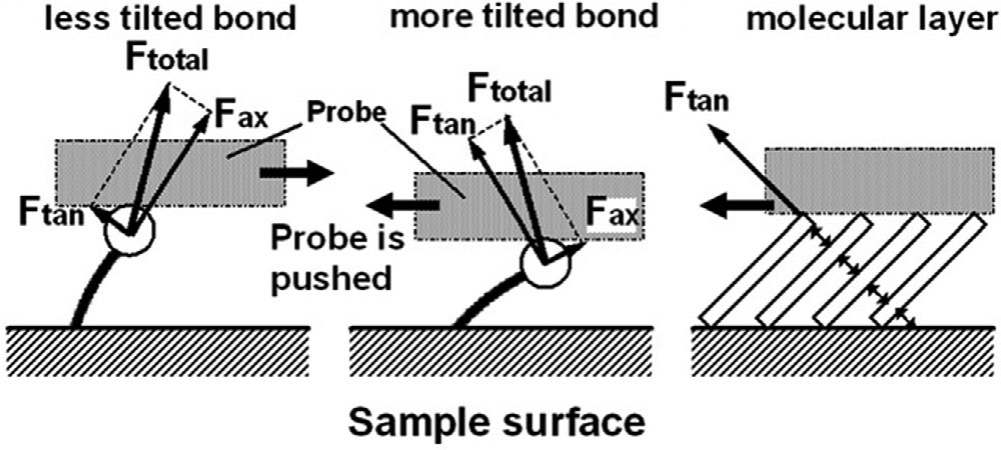
Mechanical models of interaction between the AFM probe and tilted chemical bonds modelled as mass‐spring systems. *F*_ax_ and *F*_tan_ represent the axial and tangential components of the reactive force in response to the applied normal load, respectively. The relative importance of the two components varies with the tilt angle of the chemical bonds. The direction of the horizontal component included in the total reaction may change depending on the tilt angle of the chemical bonds. When pressed by the probe, closely packed long‐chain molecules organised into a 2D molecular layer will behave like a solid due to intermolecular repulsion, yielding results akin to those observed with a more tilted chemical bond. Adapted from Ref. (98).

Between the two extreme cases of perfect commensurability and complete misalignment, the degree of atomic periodicity alignment varies continuously, leading to friction magnitudes changing with sliding direction in accordance with the material's plane symmetry.^63^ In this framework, friction can be expressed as the sum of the isotropic (direction‐independent) and anisotropic (direction‐dependent) components.^95^ This behaviour has been observed in the (001) surface of alkali halides, where the highly symmetric ionic structure allows evaluating the surface corrugation in terms of charge‐dipole interaction potential between the ions at the sample surface and a permanent dipole at the AFM tip apex. The alternating cation–anion arrangement along the [100] direction results in relatively high corrugation and friction, while along the [110], the effective alignment of cations and anions in separate rows creates a smoother potential energy landscape, reducing friction.^96^ The polar (111) surface, in contrast, exhibits consistently low and isotropic friction as it contains only one kind of ions.^64^ Ionic orientation also plays a crucial role in friction anisotropy in alkaline earth sulphate minerals such as CaSO_4_(100), SrSO_4_(001) and BaSO_4_(001).^97^ In these materials, the outermost sulphate ions are tilted to the same direction in each ionic layer,^97^ with alternating layers stuck in opposite directions. As a result, friction oscillates at monolayer steps where the surface directionality changes. The AFM probe encounters greater mechanical resistance in directions corresponding to S‐O stretching vibrations. On the layer immediately below, the AFM probe experiences less resistance due to lower force constants of S‐O bending vibrations. The highest friction occurs when scanning along the *c*‐axis, that is, against the S‐O bonds tilting in this direction.^97^ A reversed trend is observed in carbonate ions at the (10$\bar{1}$4) surface of calcite (CaCO_3_), with lower friction when scanning against the C‐O bond tilt. This may be due to the steeper tilt angle of C‐O bonds (∼53° from the normal surface) compared to the S‐O bonds tilt (∼35°). When modelling CaCO_3_ as a mass‐spring system, the recovery force of the spring for nearly tangential direction must compensate most of the normal load applied, since the axial component is geometrically less effective. Then, the total reaction will include a horizontal component which pushes the probe as indicated by the bold arrow in Figure 2.^98^ Angular dependence of the total friction on the (10$\bar{1}$4) surface shows lowest friction along the [010] direction, and highest along the [42$\bar{1}$]. Here again, similarly to alkali halides, friction anisotropy is due to the geometrical arrangement of the surface atoms. The AFM probe mainly interacts with O atoms located at higher positions than the Ca atoms; along the [010] direction, O atoms sites are spaced much more closely together than along the [42$\bar{1}$] direction. In the latter case, the tip‐sample interaction potential oscillating with a larger amplitude results in higher dissipation and hence friction.^98^, ^99^, ^100^

As a caveat, it is worth however mentioning that the variability in the shear forces experimentally measured on different surfaces of a crystalline material may be primarily dictated by changes in the contact area due to adhesion forces, with effectively no difference in the intrinsic resistance to friction.^79^ This is evident in diamond with higher friction and adhesion forces for (001) surfaces compared to (111).^79^ Additionally, the density of amorphous defects also influences preferential sliding directions and friction anisotropy in diamond. Friction anisotropy is indeed due to a complex crosstalk between inherent periodicity of the crystal lattice and the formation of amorphous defects. Frictional forces present a twofold and fourfold symmetry on the {110} and {100} planes, respectively, with amorphous defects not randomly growing but their formation being influenced by the crystal periodicity itself: the distribution of carbon atoms along <100> is sparser than along <110>, thus the tribochemical formation of amorphous defects in the latter orientation is more difficult to occur due to the limited space available.^79^, ^101^

Since contacting materials often differ, most interfaces are incommensurate.^4^ Friction anisotropy between incommensurate surfaces is observed when at least one of the surfaces is crystalline and anisotropic, meaning the periodicity of the atomic arrays at the surface changes along different directions.^102^ However, care should be taken when recording friction anisotropy using a nominally amorphous probe on a crystalline surface, as friction anisotropy may be the result of flakes of the crystalline sample being removed during scanning and stably adsorbing on the probe. This could lead to probing the motion of a crystal over itself.^92^

**FIGURE 3:**
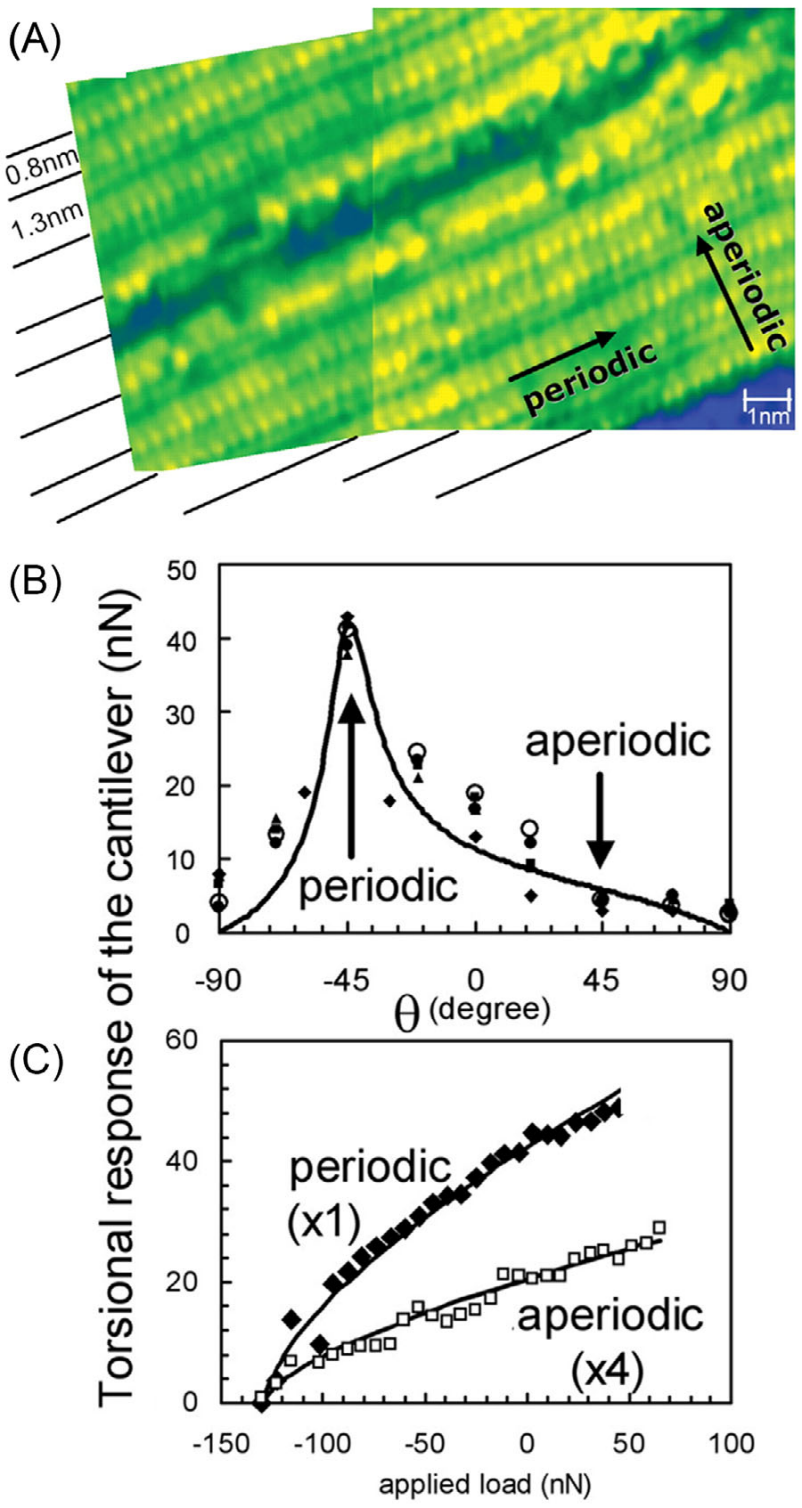
Anisotropic friction on an Al‐Ni‐Co quasicrystal surface. (A) Scanning tunneling microscopy (STM) images of the twofold Al‐Ni‐Co surface, exhibiting a 0.4 nm periodicity along the 10‐fold direction. In the direction perpendicular to the atomic rows (twofold direction), a quasiperiodic sequence of 0.8 and 1.3 nm distances is observed. (B) Torsional response of the cantilever as a function of scanning angle on the twofold surface of the Al‐Ni‐Co decagonal quasicrystal. The torsional response is higher along the periodic direction compared to the aperiodic direction. The solid line represents the calculated torsional response as a function of scanning angle, based on an anisotropy factor (friction force ratio) of 8. Data points are normalised averages of five independent measurements. (C) Torsional response as a function of applied load in both periodic and aperiodic directions. Adapted from Ref. (65).

To assess the role of periodicity in friction phenomena, an ideal approach would be to compare the tribological properties of a material in its crystalline versus amorphous states.^4^ These two states may however differ significantly chemically complicating direct comparisons.^65^ This is why quasicrystals are a valuable platform as their surfaces exhibit periodic and aperiodic atomic arrangements along different directions. For instance, in decagonal Al‐Ni‐Co quasicrystals, the twofold surface is periodic along the 10‐fold axis, but aperiodic in the perpendicular direction. Friction measured along the aperiodic direction is nearly one order of magnitude lower than along the periodic direction, though this discrepancy vanishes when the surface is oxidised^65^, ^103^, ^104^ (Figure 3). Subsequent investigations show that the anisotropy reappears after the oxide layer is worn off although cumulative wear may impact the periodicity negatively and remove any frictional anisotropy.^104^

A final remark on single crystals as model systems for friction anisotropy is related to their role in illustrating tip‐convolution effects and the surface potential at monoatomic step sites.^75^, ^76^, ^77^, ^105^, ^106^ Geometric effects can influence the direction dependence of dissipation channels, with higher friction observed when scanning perpendicular to the steps compared to parallel.^76^ Further mechanisms are however behind the recorded friction asymmetry, deriving from a different friction related to the trace and retrace sliding of the probe over the same scan line. Friction is indeed found greater when scanning upwards over a step compared to downwards, likely due to the Schwöebel‐Ehrlich barrier^76^ where the adsorption energy and interaction potential for a tip approaching the step edge are higher because of the greater number of nearest neighbours compared to an atom on the terrace.^75^, ^76^, ^77^ The association between increased friction at step edges and the presence of a Schwöbel–Ehrlich barrier appears to hold primarily for blunt tips, where strong repulsive interactions between the tip apex and surface atoms are inevitable, often leading to abrasion wear. In contrast, an atomically sharp tip can traverse step edges without encountering a significant activation barrier or inducing surface wear. Achieving this requires only a low applied load, near the threshold for tip detachment, that is, close to jump‐off. Notably, the ability of a sharp tip to eliminate the enhanced friction observed during upward scans appears to be a general phenomenon. This effect has been demonstrated not only on ionic crystals like NaCl(001) but also on semiconducting surfaces such as Ge(001).^107^

### 2.2 2D materials

**FIGURE 4:**
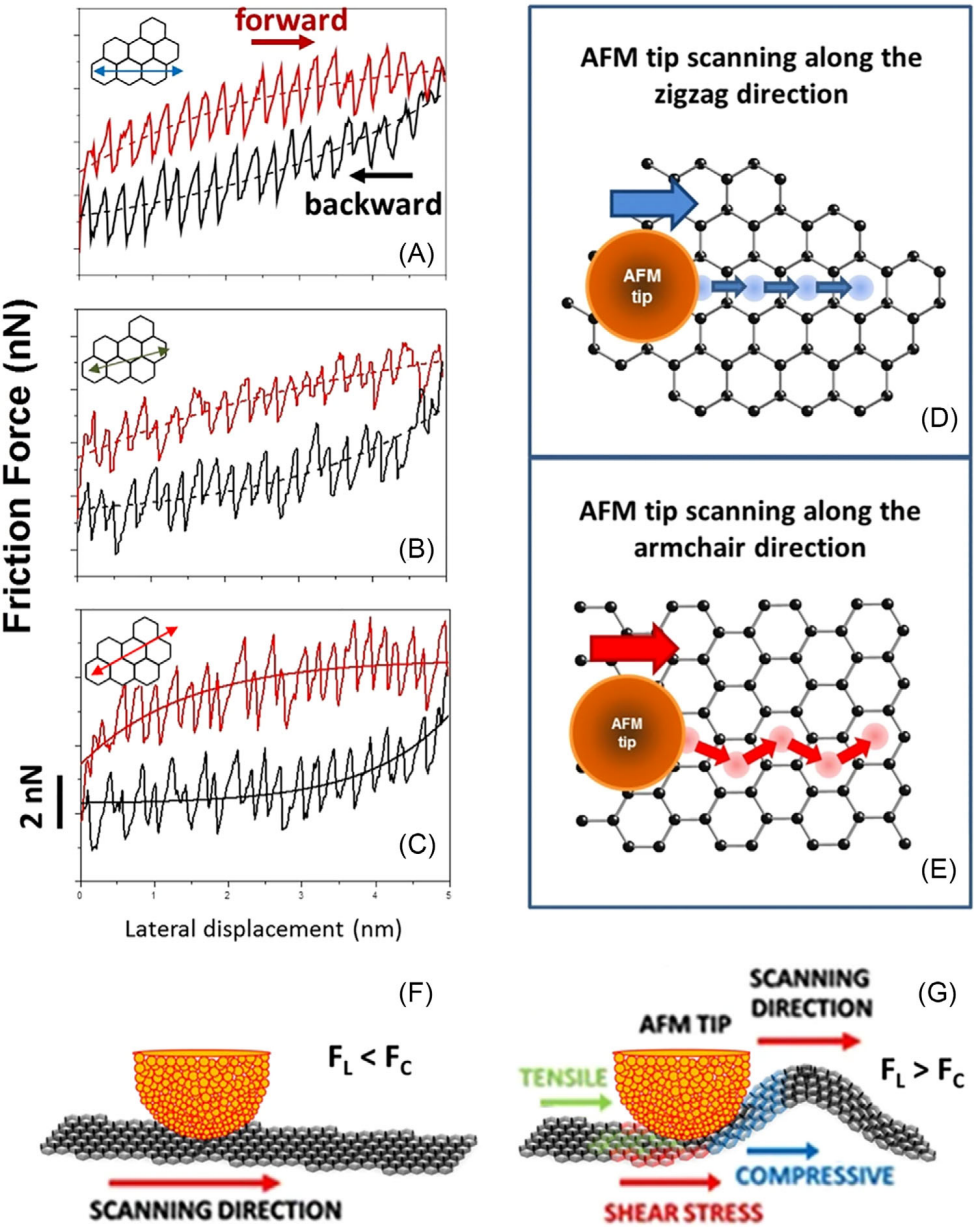
Friction forces in graphene. (A–C) Friction force values as a function of the tip's lateral displacement along the zigzag (A), 15° off zigzag (B), and armchair (C) directions. The dashed lines represent exponentially saturating functions that illustrate the strengthening and saturation of friction forces. Insets depict the tip scanning directions over the graphene sheet. (D, E) The spatial distribution of local minima in the tip‐graphene interaction potential along the zigzag and armchair directions, respectively. Blue and red circles within the hexagonal lattice of graphene represent sticking points at local potential minima for the zigzag and armchair directions, respectively. Arrows indicate slip jumps. Along the zigzag direction, all jumps are equal, giving the corresponding force profile a simple sawtooth shape. Along the armchair direction, the jumps form a zigzag pattern, resulting in a force profile with a double‐period structure. For arbitrary directions, the force profiles are more complex. (F) When lateral forces *F_L_* are smaller than the critical force *F_c_* for Euler buckling, the graphene sheet remains planar. (G) In contrast when the lateral forces exceed the critical force, flexural deformations (buckling) occur. Adapted from Ref. (125).

Lamellar (layered) crystals, which can be prepared via mechanical cleavage, epitaxial growth or chemical vapour deposition (CVD), provide valuable experimental models for crystalline surfaces. These processes enable the preparation of atomically or molecularly flat surfaces, also known as 2‐dimensional (2D) materials.^40^, ^91^, ^108^, ^109^, ^110^, ^111^, ^112^, ^113^, ^114^, ^115^, ^116^, ^117^, ^118^, ^119^, ^120^, ^121^, ^122^, ^123^, ^124^ Among these, highly ordered pyrolytic graphite (HOPG) holds a prominent position: exfoliating this material allows exploration of a broad spectrum of dimensionality ranging from classical 3D down to the single layer (i.e. graphene)^110^, ^113^, ^114^, ^115^, ^116^ with applications spanning supercapacitors,^117^, ^118^ thermo‐electrics,^114^, ^119^ catalysis^122^ and solid lubricants.^10^, ^91^, ^123^, ^124^ In the basal plane of HOPG, two directions with a periodicity of 60° (sixfold symmetry) can be distinguished: the zigzag and armchair axes.^67^, ^125^, ^126^, ^127^, ^128^, ^129^, ^130^, ^131^ The armchair direction represents the high friction direction^125^, ^126^, ^127^, ^128^ (Figure 4A–E). Frictional anisotropy of HOPG, measured by lateral force microscopy (LFM), is around 15%. Interestingly, in single layer graphene, this anisotropy is amplified and load dependent, reaching approximately 80%. This behaviour has been attributed to Euler buckling, where tip‐induced out‐of‐plane (flexural) deformations of the graphene sheet^125^, ^132^ are isotropically amplified. As a result, friction forces and anisotropy increase when the lateral force exceeds a critical threshold and as sliding distance grows^125^, ^133^, ^134^ (Figure 4F and G).

Similar frictional anisotropy is observed in other 2D hexagonal lattice materials such as molybdenum disulphide (MoS_2_) or hexagonal boron nitride (*h*‐BN). These materials exhibit two in‐plane inherent lattice orientations: the armchair and the zigzag directions. The anisotropy originates from differences in the periodic potential energy landscape experienced by the AFM tip along the armchair and zigzag directions, which modify the stick–slip dynamics and energy dissipation during sliding. ^67^, ^135^

Friction anisotropy related to deformation and dissipation along the armchair and the zigzag directions has also been identified in other 2D materials with non‐hexagonal crystal lattices, such as black phosphorous which has an orthorhombic structure.^67^, ^120^, ^136^, ^137^ The zigzag direction provides a preferential sliding axis when a non‐2D material is placed on top of a 2D material, as demonstrated by gold nanoislands on MoS_2_, which exhibits a periodicity of 60°.^138^ Similarly, a periodicity of 60° for commensurate contacts is found when sliding carbon nanotubes on HOPG.^139^

Puckering effects also influence the friction response in MoS_2_ and MoSe_2_ depending on their thickness.^135^, ^140^, ^141^, ^142^, ^143^ In MoS_2_, decreasing the sample thickness from 45.23 nm (tens of layers) to 4.18 nm and then to 1.49 nm (still larger than the thickness of a single layer ~0.8 nm)^144^ results in two key effects: friction increases with load along both the zigzag and the armchair directions, but friction anisotropy decreases.^135^ These effects are closely related: thinner samples exhibit a larger contact area due to the reduced bending stiffness, which enhances out‐of‐plane deformation (contact area) between the AFM probe and MoS_2_. Moreover, friction anisotropy weakens with increased deformation along both zigzag and armchair directions; and, the puckering effect is suppressed in thicker samples, with friction anisotropy governed primarily by lattice orientation.^135^ Further studies on mono‐ and few‐layer MoSe_2_ flakes deposited on sapphire confirm that friction increases as sample thickness decreases, likely due to out‐of‐plane deformation.^140^ Similar to MoS_2_,^135^ friction anisotropy in MoSe_2_ is caused by different dissipation channels depending on sample thickness.^140^ For few‐layers, friction is predominantly governed by atomic‐induced stick–slip motion, while for monolayer flakes, friction anisotropy arises from the interplay between out‐of‐plane deformation and stick–slip motion.^140^ Interestingly, the magnitude of friction anisotropy does not significantly change between mono‐ and few‐layer MoSe_2_,^140^ contrasting with the findings for MoS_2_.^135^

A further thickness‐related effect observed in MoSe_2_, differing from MoS_2_,^135^ is the change of preferential sliding direction.^140^ For mono‐layer MoSe_2_, friction maxima occur along the zigzag direction, whereas for few‐layers samples they occur along the armchair direction. In both cases, the sixfold symmetry is conserved and friction forces measured in the high‐friction direction are enhanced by ∼ 60% compared to the low‐friction direction.^140^ The anisotropy observed in mono‐layer MoSe_2_ is consistent with other 2D transition‐metal dichalcogenide (TMD) monolayers (i.e., WS_2_), which exhibit lower friction along the armchair orientation.^142^ Interestingly, chalcogen substitution in 2D TMDs has been shown to have a key role in influencing friction and hence friction anisotropy, by modifying the interplay between the tip‐sample interfacial energy barrier and the lattice constant.^143^

However, the sixfold symmetry^125^, ^126^, ^145^, ^146^ can be disrupted because of out‐of‐plane elastic deformation caused by linearly oriented wrinkles, resulting in a twofold symmetry with higher friction when scanning perpendicularly to the wrinkles.^129^, ^131^, ^135^, ^147^ This presents a common experimental challenge when probing 2D materials: depositing them on a classical 3D substrate induces stresses at the adlayer/substrate interface.^132^, ^134^, ^135^, ^148^, ^149^, ^150^ These stresses relax through inhomogeneous strain fields, leading to the formation of atomic and nanoscale corrugation such as atomic defects,^80^ linearly aligned ripples^131^, ^151^, ^152^, ^153^ and wrinkles.^145^ In HOPG, defects can also be engineered via anion intercalation through electrochemical methods.^154^ Ripples are widely observed in 2D van der Waals materials.^66^, ^116^, ^147^, ^153^, ^155^, ^156^ Overall, all the different types of periodic corrugation contribute to friction anisotropy when an AFM probe slides over them.^80^, ^131^, ^135^, ^145^, ^147^, ^151^, ^157^ For instance, in graphene, high friction occurs when the AFM tip slides perpendicular to the ripple crests and low friction is observed when sliding parallel.^151^ Although all types of periodic surface corrugation typically produce similar frictional anisotropy effects, they differ in length scale. The periodicity ranges from 0.1–0.5 nm for atomic corrugations^80^ to 5–100 nm for grooves^158^ and ripples,^131^, ^159^ with wrinkles reaching the microscale.^145^ Interestingly, periodic ripples also induce friction anisotropy in other materials, such as Ar^+^ ion beam irradiated silicon surfaces.^160^

The impact of surface corrugation on friction anisotropy is further confirmed by chemical modifications that alter the substrate's periodicity. For example, helium ion irradiation can increase defects density in CVD grown single layer MoS_2_ significantly enhancing friction, even though MoS_2_ maintains its 2D nature at nanoscale.^80^ Fluorination of graphene layers is another striking example of chemically induced friction tuning: friction can increase 5–9 times compared to pristine graphene.^161^, ^162^, ^163^ Two mechanisms are proposed: the fluorination modifies the interfacial potential due to strong local charges at fluorine sites, with friction increasing as a function of surface corrugation,^163^ and with some impact also on anisotropy.^164^ Otherwise, fluorination of graphene would increase up to 4 times the out‐of‐plane bending stiffness of the material making it less compliant, thus increasing dissipation while slightly reducing adhesion forces.^162^

In addition to chemical modifications, mechanical strain by epitaxial growth is another effective way to modify the properties of thin films and crystals.^165^ For instance growing centrosymmetric MoS_2_ bilayers on a MoS_2_ monolayer creates star‐shaped strain patterns in one‐dimensional nanoripple arrays, doubling the friction primarily due to the large elastic deformation of the nanoripples.^166^ Friction anisotropy due to puckering‐induced defects and surface corrugation extends beyond 2D materials. For example, grooves on atomically flat mineral surfaces also lead to friction anisotropy with two orthogonal symmetry planes where friction is high (low) when scanning perpendicular (parallel) to the grooves.^158^ Strain from interlayer interactions can also destroy friction anisotropy in 2D materials. Using Ti_3_C_2_T*_x_* MXenes deposited on silicon dioxide as a model system, friction anisotropy with sixfold symmetry is reduced with increasing number of atomic layers, disappearing when the number of layers exceeds 10.^167^ This suggests that as the number of layers increases, the binding energy between them strengthens, preventing Euler buckling.

Recent experiments have further examined how strain and anisotropic suspension of 2D materials influence friction anisotropy. Clamping a single graphene layer across a long, narrow groove results in significant anisotropy with respect to the groove axis. A low COF (∼0.005) is measured when sliding perpendicular to the groove while a nearly threefold increase occurs when sliding parallel. The lack of pre‐strain and the deformation induced by the indentation and sliding action of the tip causes the friction anisotropy due to the asymmetric clamping conditions. Sliding orthogonally to the groove generates more strain, causing the graphene membrane to stiffen, and reducing the force needed to slide the tip. In contrast, sliding parallel to the grooves allows the graphene membrane to easily deform, requiring more force to slide the tip. This mechanism becomes more pronounced with increasing load, which results in asymmetric dependence of friction versus load.^168^

**FIGURE 5:**
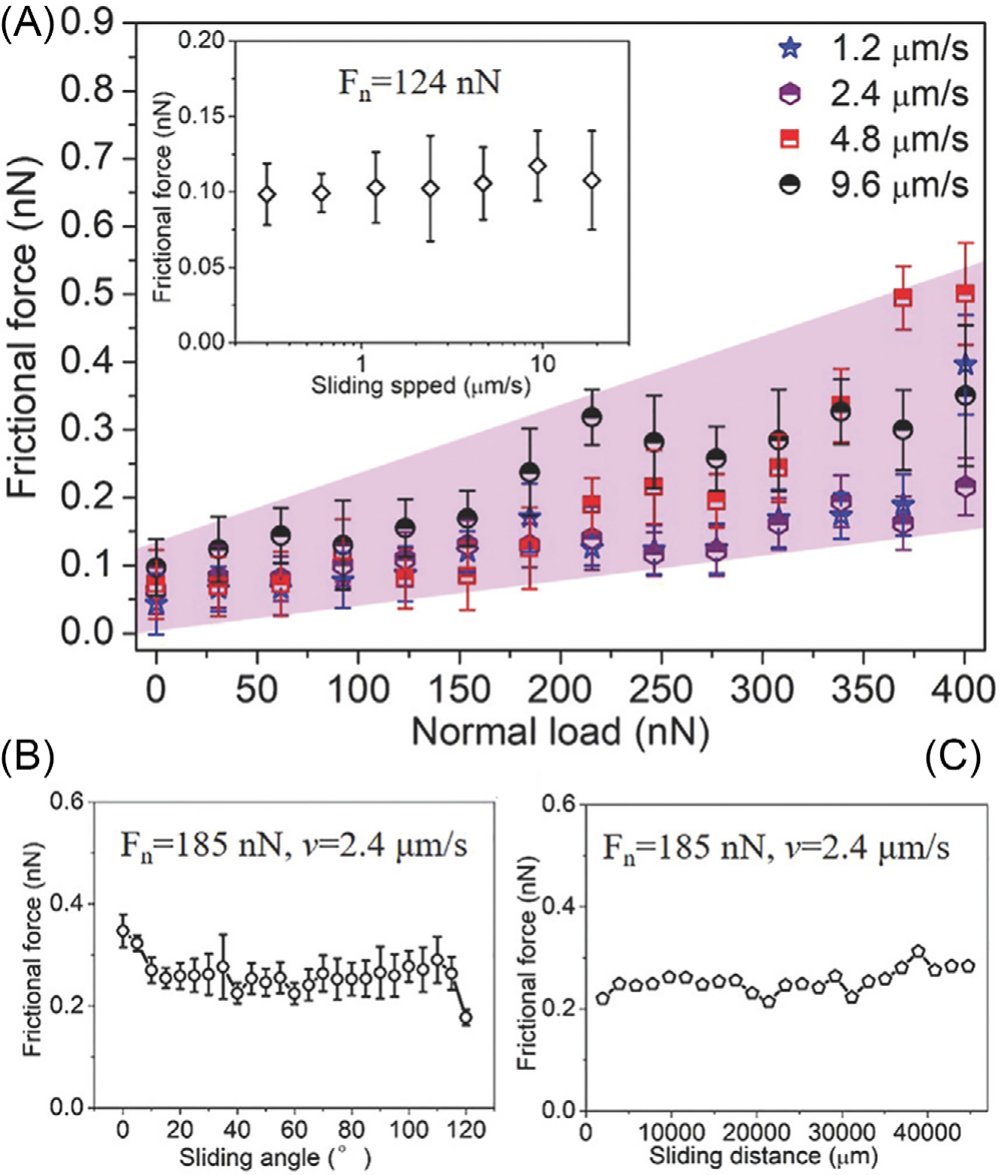
Frictional force between silica particles glued to an AFM probe and a HOPG substrate. (A) Frictional force is plotted against normal load at four different sliding speeds (1.2, 2.4, 4.8, and 9.6 µm/s). The inset shows the relationship between the frictional force and sliding speed at a constant normal load of 124 nN. (B) Relationship between frictional force and sliding angle (varied from 0° to 120°) at a constant normal load of 185 nN and a sliding velocity of 2.4 µm/s. (C) Relationship between frictional force and sliding distance (measured continuously) at a constant normal load of 185 nN and a velocity of 2.4 µm/s. Adapted from Ref. (169).

Notably, twofold symmetry in 2D materials can also arise from variations in the tip‐sample contact quality, specifically the strength of the atomic interactions between the tip and sample. For this mechanism to significantly affect interfacial energy dissipation, and thus friction, the AFM probe should typically scan atomically flat regions only a few nanometers across, i.e., smaller than the characteristic size of the surface defects.^129^ The importance of tip‐sample contact quality is also supported by experiments where silica particles, glued to an AFM probe, were slid across an HOPG substrate.^169^ After an initial pre‐sliding, graphene nanoflakes (GNFs) adhered to the silica particle asperities, shifting the shear plane from silica/graphite to graphene/graphite due to the lower shear strength of this interface. Since the lattices of the GNFs and HOPG are perfectly aligned and rigid, the resulting incommensurate contact between the asperities and the substrate led to robust, angle‐independent superlubricity (Figure 5).^169^

It is also noteworthy that some studies suggest an alternative mechanism behind the observed twofold symmetry in graphene.^170^ In this material, periodic ripples typically result in high friction along the axis perpendicular to the stripes^131^, ^151^ and the zigzag stripe axis.^156^ When this behaviour is not observed, friction anisotropy may arise from airborne contaminants that stably adsorb and self‐assembly on the surface,^170^, ^171^, ^172^ forming regular stripe‐like features, instead of periodic ripples.^170^

**FIGURE 6:**
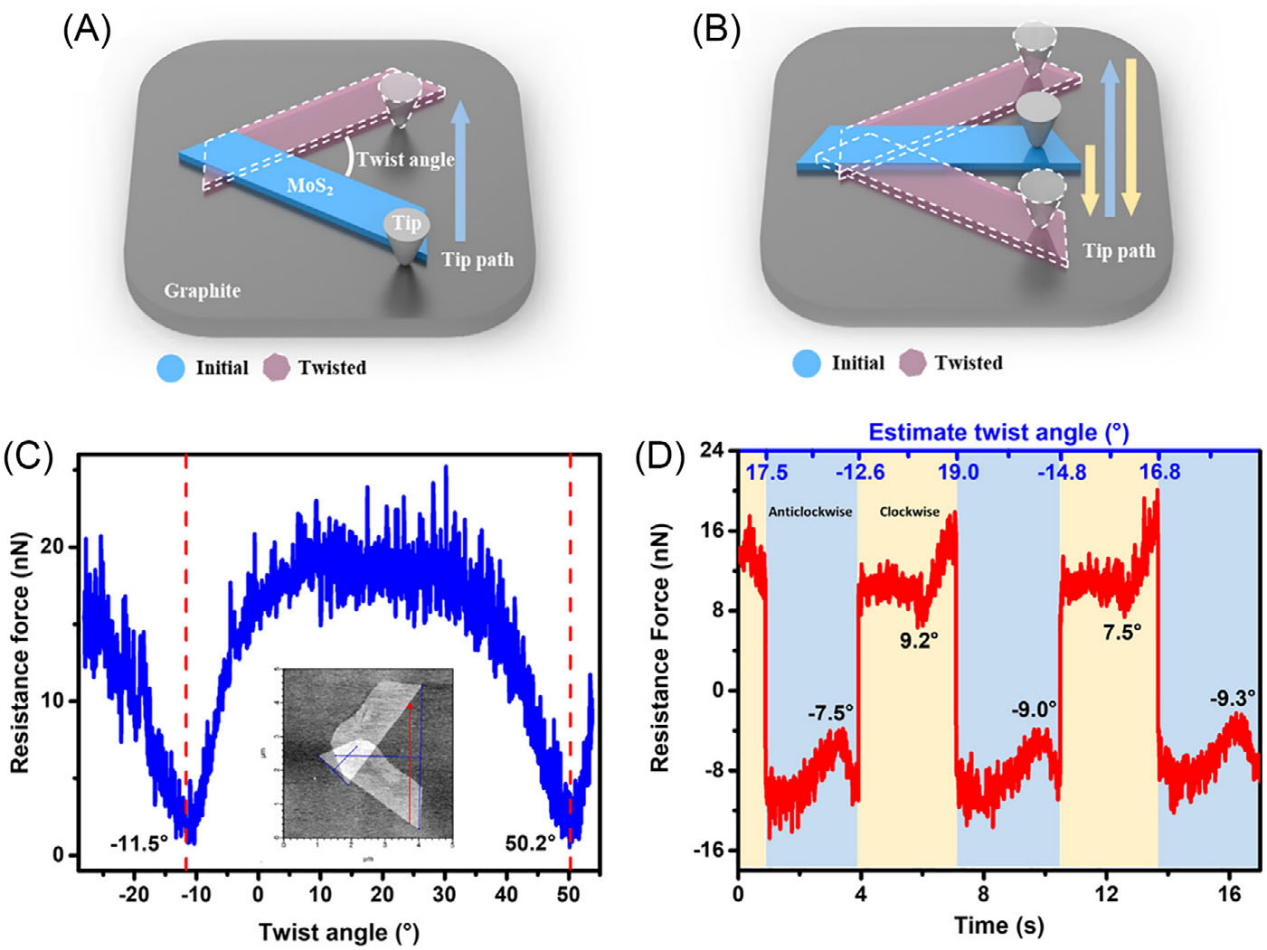
Dynamic rotational resistance force measurements of the MoS_2_/graphite heterostructure. The rotational dynamics of the system was investigated using two different AFM probe sliding methods: the ‘push the edge’ method (A) and the ‘drag the centre’ method (B). In the ‘push the edge’ method, the AFM tip moves perpendicular to the cantilever beam direction, pushing the edge of the MoS_2_ domain. The centre and angle of rotation are determined by overlapping the AFM images before and after displacement. In the ‘drag the centre’ method, the AFM tip first engages the middle of one side of the MoS_2_ domain, then moves back and forth perpendicular to the cantilever beam direction to drag the MoS_2_ domain. For the ‘push the edge’ method, due to the ultralow sliding friction in the MoS_2_/graphite heterostructure, the translational energy barrier is lower than the rotational energy barrier, leading to MoS_2_ domains to favour rigid translation over twisting. However, pure translation can be hindered by structural damage, the presence of a fulcrum, or air exposure. The ‘drag the centre’ method minimises damage, allowing controlled rotation around a central point. As shown in (C, D), both methods, however, show periodic rotational resistance. (C) Rotational resistance force of the MoS_2_/graphite heterostructure as a function of the twist angle, measured by the ‘push the edge’ method. The inset shows overlapping AFM images before and after rotation. (D) Clockwise and anticlockwise rotational resistance force of the MoS_2_/graphite heterostructure, measured by continuous motion in both directions using the ‘drag the centre’ method. Adapted from Ref. (181).

Interestingly, 2D materials can be combined into homo‐ or heterojunctions, enabling the design of incommensurate interfaces with superlubric characteristics.^94^, ^173^, ^174^, ^175^ For example, a superlubric system based on a 2D homojunction can be engineered with a nanoscale graphene‐coated corrugated sphere sliding atop flat graphite.^176^ Although such homojunctions can provide superlubric interfaces, their small area leads to extremely high local pressures, which may cause enhanced wear impacting the system durability. To overcome this limitation, heterogeneous junctions with extended single‐crystalline surfaces have been proposed as an ideal system for structural superlubricity. These junctions are robust even against crystal reorientations and have improved durability. This is because the intrinsic lattice constant mismatch between contacting surfaces creates incommensurability at any twist angle, even when the lattice vectors of the two surfaces are aligned.^177^ Experimental studies on pristine microscale heterojunctions between single crystalline *h*‐BN and graphite have confirmed this hypothesis showing that structural superlubricity persists even when the aligned contact sustains external loads under ambient conditions. Additionally, the frictional anisotropy in these heterojunctions is orders of magnitude smaller than that observed in their homogeneous counterparts.^177^, ^178^ When aiming for twist‐angle independent superlubricity or ultra‐low friction, a key consideration is the degree of lattice mismatch within the interface. For instance, in the interface between graphene and *h*‐BN, the lattice constants differ by approximately 2%,^94^ which can give rise to a moiré superlattice^179^, ^180^ that develops near zero angle mismatch resulting in non‐negligible frictional forces when the layers are aligned.^179^ However, by using a combination of 2D interfaces with lattice mismatch larger of over 20% (e.g. MoS_2_/graphite, MoS_2_/*h*‐BN), twist‐angle independence and ultralow COF of the order of 10^−6^ can be achieved^94^, ^181^ – well below the threshold for superlubricity (COF<10^−3^).^4^, ^94^ For MoS_2_/graphite heterostructures, even in the presence of twist‐angle independent superlubric sliding, the evolving potential energy change can induce a non‐zero rotational resistance force which valleys at a 60° period, making the system rotationally asymmetric.^181^ This occurs because the structural changes in the heterostructure during twisting introduce additional pathway for energy dissipation: as the heterostructure twists, continuous changes in the moiré superstructure and interlayer distance lead to in‐plane and out‐of‐plane vibrations, transferring part of the energy to heat. In the rotational motion of 2D heterostructures, even if the interface remains incommensurate and structural lubricity is maintained, the potential energy change can directly modify the friction force (e.g.through the edge‐pinning effect), resulting in a periodic rotational resistance force (Figure 6).^181^

Before assembling 2D materials into homo‐ and heterojunctions, controlling the crystallographic orientation on a substrate enables the assessment of chemical bonding contribution to friction anisotropy.^182^, ^183^ Model systems of MoS_2_ or PtSe_2_ layers are deposited on sapphire substrate with their *c*‐axis oriented either in‐plane or out‐of‐plane. These 2D materials are TMDs and consist of two planes of chalcogen atoms surrounding an interstitial plane of transition metal atoms.^182^  For instance, in MoS_2_, each fundamental layer comprises a Mo atom sheet sandwiched between two S atom sheets. While strong covalent bonds bind Mo and S atoms within each layer, adjacent layers interact via weak van der Waals bonds, with the crystallographic *c*‐axis perpendicular to the layers.^183^ When the *c*‐axis is aligned parallel to the surface, the dominance of weak van der Waals interactions leads to a lower coefficient of friction compared to vertically aligned sheet.^182^, ^183^ Interestingly, epitaxial films show even lower friction due to reduced adhesion.^182^

The use of layered materials as model surfaces has significantly advanced our understanding of commensurability. For instance, MoO_3_ islands on a MoS_2_ substrate can be displaced by an AFM probe along preferred directions dictated by the alignment of the MoO_3_ and MoS_2_ crystal axes.^112^, ^184^, ^185^ Recent experimental and computational studies on amorphous and crystalline MoS_2_ sliding against themselves reveal that commensurability is not solely determined by the outermost surface atoms of the two contacting materials.^4^, ^186^ Instead, it must also account for interlayer commensurability within each material. In the most dissipative scenario – where layers within each material are perfectly aligned – friction is maximised. Furthermore, commensurability is influenced by the chemical nature of the two contacting surfaces. For example, for two commensurate crystalline MoS_2_ surfaces, where surface atoms primarily interact by van der Waals forces, sliding is energetically more favourable than in amorphous MoS_2_ over amorphous MoS_2_. In the latter case, friction is higher due to the need to overcome van der Waals forces and continuously break and reform covalent bonds caused by unsaturated surface atoms. This explains why crystalline MoS_2_ exhibits a lower friction coefficient compared to its amorphous counterpart.^186^ The role of chemical interactions and contact area in friction for nominally incommensurate surfaces has been further explored in a combined experimental and theoretical study sliding Sb particles on two layered materials, HOPG and MOS_2_.^187^ On HOPG, sliding behaviour aligns with the concept of structural superlubricity, whereas on MoS_2_, a transition from superlubricity to constant shear stress occurs beyond a certain particle size. This shift is attributed to the formation of dislocations, which act as dissipation channels in large Sb particles, disrupting structural superlubricity.^187^, ^188^ Additionally, oxidation and ambient contaminants have been shown to affect Sb particle motion over HOPG in vacuum, emphasising the importance of effective contact area and adsorbed molecules in friction anisotropy.^189^, ^190^

The broad applicability of 2D materials in friction anisotropy is further demonstrated by a study of graphene sliding on Pt(111) surfaces. Here, surface periodicity is again the key factor influencing friction, with frictional forces modulated by the moiré pattern periodicity arising from the orientation‐dependent mismatch between graphene and Pt(111).^191^

### 2.3 Organic molecular crystals and organic salts

**FIGURE 7:**
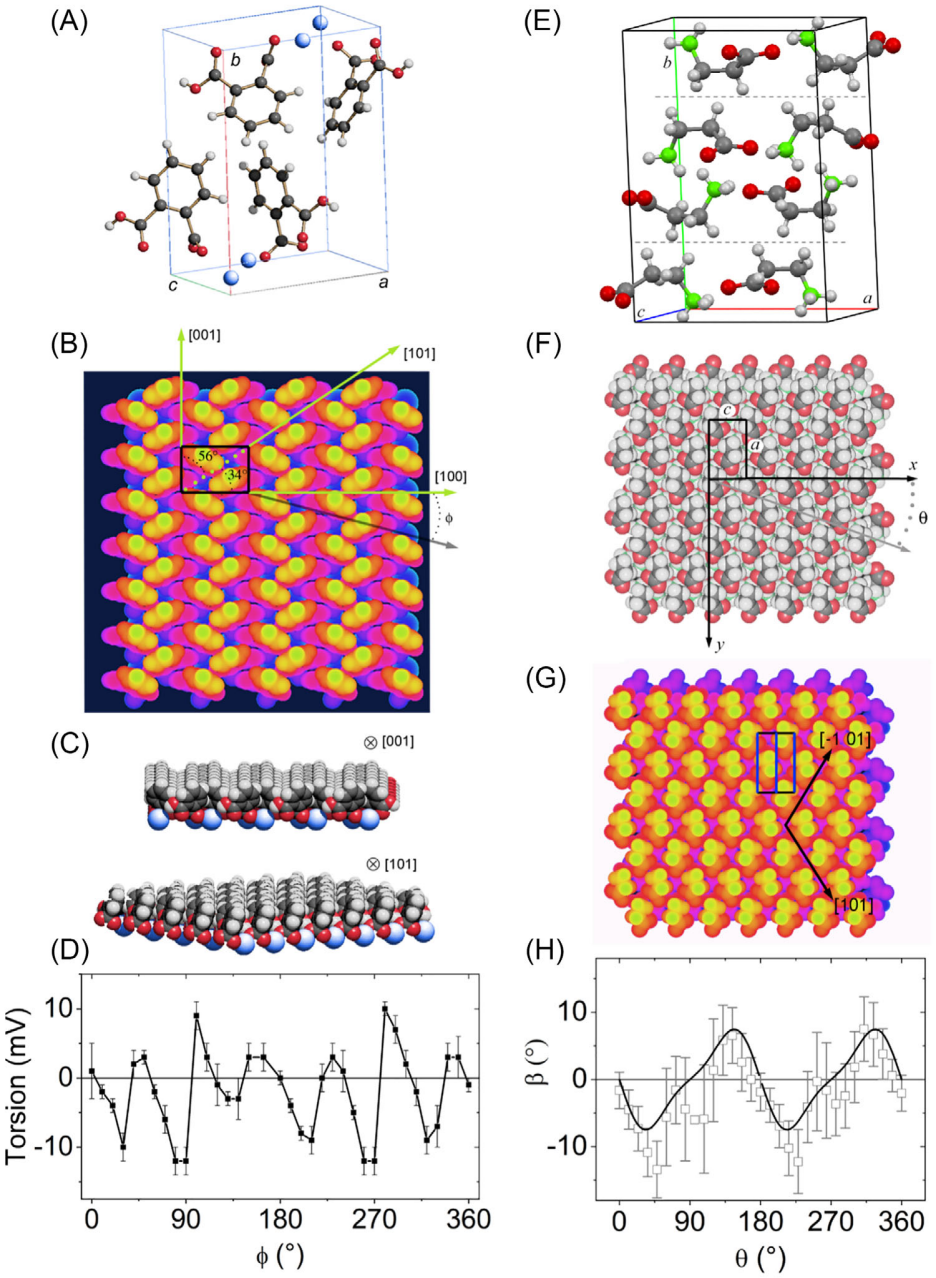
Structural origins of friction anisotropy on KAP and β‐alanine crystal surfaces. (A) Crystal structure of KAP [grey (large dark spheres): C‐atoms; red (small dark balls): O‐atoms and ions; blue (large bright spheres): K‐ions; white (small bright spheres): H‐atoms] and unit cell axes. Unit cell parameters *a* = 9.61 Å, *b* = 13.33 Å, *c* = 6.48 Å. (B) Depth‐dependent colour representation of the cleavage surface of KAP with the surface unit cell outlined by a black rectangle. Significant crystallographic directions are indicated by green (bright) arrows; *φ* represents the angle between KAP[100] and the scan direction (black arrow). (C) Structural models of the cleavage surface of KAP, viewed along the [001] and [101] directions, highlighting key surface corrugations. (D) Experimental torsion signal measured on the KAP (010) surface as a function of the angle φ (in 10° increments) with the tip scanning along a direction parallel to the cantilever axis. Adapted from Ref. (193). (E) Crystal structure of β‐alanine; light grey: C‐atoms; red (dark grey): O‐atoms; green (large white spheres): N‐atoms; white (small spheres): H‐atoms; the dashed lines indicate the orientation and position of the (010) growth surfaces. Unit cell parameters: *a* = 9.88 Å, *b* = 13.81 Å, *c* = 6.09 Å. (F) Model of the (010) growth surface of β‐alanine, including the Cartesian reference frame for the AFM measurements. The surface unit cell is marked by a black rectangle. During scanning, the cantilever axis remains parallel to β‐alanine [001] while the tip slip direction (grey arrow) forms an angle *θ* (scan angle) with β‐alanine[001]. (G) Depth‐dependent colour model for the (010) growth surface of β‐alanine. The plane symmetry corresponds to the *Pg* group, where only glide planes (marked as vertical blue segments within the unit cell) are allowed symmetry elements. (H) Angle *β*, representing the deviation between the friction force vector and the slip vector, as a function of the slip direction, derived from friction hodograph measurements. Adapted from Ref. (194).

Compared to 2D materials, the study of surface corrugation and frictional anisotropy/asymmetry in organic molecular materials remains relatively limited. Some investigations have however focused on the cleavage surfaces of L‐alanine,^192^ orthorhombic potassium acid phthalate (KAP)^193^ (Figure 7A) and β‐alanine^194^ (Figure 7E). In KAP and β‐alanine, the surface molecular arrangement gives rise to ridges and grooves along specific crystallographic directions (Figure 7B,C,F, and G). AFM friction force measurements show that sliding parallel to the grooves results in significantly lower friction than sliding perpendicular to them, with no transverse force component in either parallel or perpendicular motion. However, in other slip directions, a tip torsion occurs, indicating the presence of transverse friction components (Figure 7D and H). The principal directions of friction – where sliding occurs without transverse friction components – are determined by a combination of surface molecular structure and crystallographic symmetry. Thus, even crystals with similar crystal symmetry or comparable lattice dimensions may exhibit a different number of principal friction directions due to differences in molecular packing and surface interactions. For example, the KAP(010) surface has six principal directions (Figure 7D), whereas the β‐alanine(010) surface has only two (Figure 7H).

Prominent benzyl groups arranged in a herringbone motif similar to that observed on the KAP(010) surface (Figure 7B), also define the layered structure of the salt bis(benzylammonium)bis(oxalate)cuprate(II)^195^ and the organic semiconductor pentacene (C_22_H_14_).^196^, ^197^ On the former, high‐resolution AFM imaging reveals a crystallographically anisotropic molecular surface, where molecular rows adopt distinct orientations leading to friction anisotropy.^195^ Similarly, in pentacene, the observed friction anisotropy arises from both the molecular orientation and elastic anisotropy, with friction peaking along the [110] direction, reaching its lowest values along [$\bar{1}$10], and showing intermediate values along [100] and [010].^197^ Organic semiconductor crystals incorporating cyano‐vinylene moieties provide another example of how molecular organisation modulates direction‐dependent compliance, resulting in anisotropic frictional forces.^198^

Molecular structure and ordering are behind friction anisotropy also in organic semiconductor crystals. In (2Z,2′Z)‐3,3′‐(1,4‐phenylene)bis(2‐(4‐butoxyphenyl)acrylonitrile) (β‐DBDCS) crystals, friction forces strongly correlate with the anisotropic packing of molecular chains along the [010] and [001] directions. Sliding along [001] leads to higher friction due to closely packed alkyl chains and ‘steric constriction’, whereas sliding along [010] results in lower friction. This anisotropy also affects wear resistance, with the maximum applicable normal force increasing by a factor of three when scanning along [001].^199^ It is important to note that in organic semiconductor crystals, molecular ordering evolves with ageing, a process that can be accelerated through thermal annealing. This structural evolution impacts frictional anisotropy, with friction forces varying based on whether the local film state is in a metastable monolayer phase or a stable bilayer‐type herringbone phase. The latter, characterised by densely packed and smooth alkyl chain layers, is less deformable and thus less dissipative than the more disordered self‐assembled monolayer (SAM) systems.^200^

Reducing surface symmetry is a strategic approach for designing technological substrates that induce uniaxial alignment of deposited functional overlayers. Uniaxiality is crucial for minimising extended defects such as grain boundaries and enhancing performance in the case of strong anisotropic response.^201^ This strategy can be implemented by using purposely designed molecular crystals. A notable example is the mixed crystal of fumaric acid and 2,5‐diketopiperazine 1:1 which possesses triclinic symmetry and a lamellar structure enabling exfoliation along the (110) plane. This chiral surface has been demonstrated to induce the uniaxial alignment of other molecular overlayers.^84^, ^202^

The interface between organic crystals and 2D materials has provided valuable insights into how epitaxial growth and epitaxial relationships influence friction anisotropy. This has been demonstrated using epitaxial *para*‐hexaphenyl crystallites grown on graphene and *h*‐BN. The epitaxial locking of the corrugated interface between the molecular crystal and the substrate constrains crystallite sliding to preferential directions which correspond to the growth directions of crystallites. In this model system, friction anisotropy arises due to the registry between the crystallite and the substrate. Friction is high when the crystallite is in registry with the substrate (i.e. in a rotationally commensurate relationship) and low when the crystallite is out of registry (i.e. in a rotationally incommensurate relationship).^203^

Beyond epitaxial effects, organic crystals can also exhibit friction anisotropy due to their ferroelectric properties, offering a unique platform to control friction via an electric field and vice versa. For example, the (010) cleavage face of ferroelectric triglycine sulphate (TGS) single crystals exhibits direction‐dependent friction, driven by the geometrical arrangement of molecules. This arrangement dictates the surface potential, thereby modulating the friction coefficient. In other words, friction anisotropy in this system is coupled to ferroelectric domains of different polarity. Additionally, the TGS (010) surface displays an asymmetric friction response to applied load due to the asymmetric molecular arrangement within each domain.^204^

## 3 THE ROLE OF ADSORBATES

Friction anisotropy can also emerge from the presence of solid or liquid adsorbates on sliding surfaces. In many practical applications, lubrication is achieved using either solid particles,^123^, ^205^ or, more commonly, liquid lubricants.^4^, ^206^, ^207^ Understanding the role of confined molecules between sliding surfaces is therefore crucial from both a fundamental and technological perspective.

### 3.1 Solid adsorbates

**FIGURE 8:**
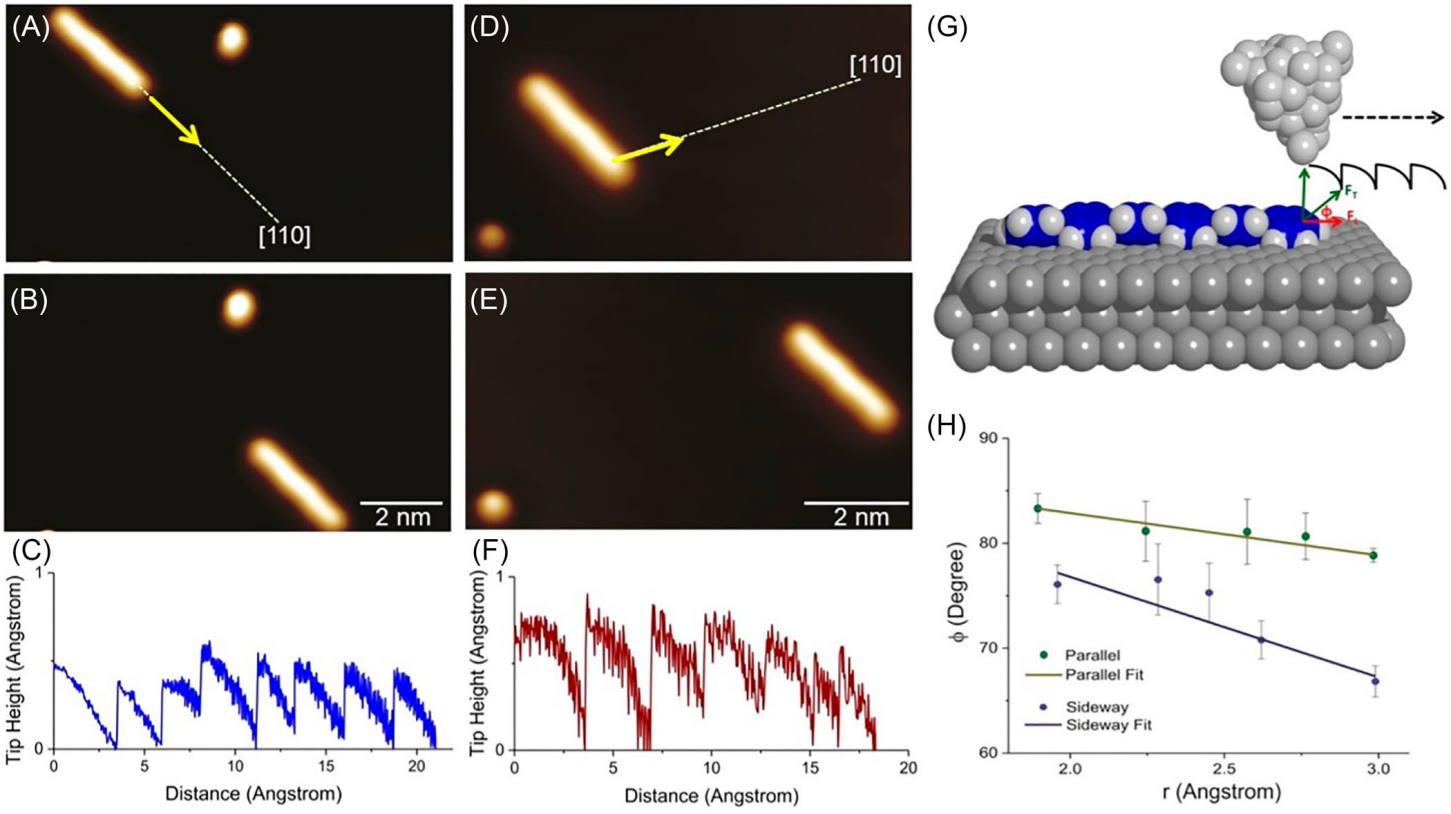
STM manipulation of 6P molecules: directional dependence of lateral forces. STM image of 6P molecule before (A) and after lateral manipulation (B) along a direction parallel to its long molecular axis (indicated by an arrow). Image parameters for (A, B): 14.5 × 6.3 nm^2^, *I*_t_ = 1.3 × 10^−9^ A, *V*_t_ = 0.42 V. (C) A typical pulling manipulation curve recorded during manipulation along the parallel direction. (D, E) STM images of a 6P molecule before and after lateral manipulation along a sideways direction (indicated by an arrow). Image parameters for (D, E): 11.4 × 4.9 nm^2^, *I*_t_ = 1.3 × 10^−9^ A, *V*_t_ = 0.42 V. (F) A typical pulling manipulation curve recorded during manipulation along the sideways direction. Manipulation parameters for (C, F): *R*_t_ = 1.2 MΩ, *V*_t_ = 0.12 V. (G) Schematic representation of the rest‐hop motion of the 6P molecule during STM‐tip manipulation, producing a characteristic pulling manipulation signal (black curve). The total and lateral forces (*F*_T_ and *F*_L_) and the force angle *ϕ* are illustrated. (H) Force angle as a function of tip height for both parallel and sideways directions. The error bars describe the mean statistical distributions, while the straight lines indicate linear fits. From Ref. (209).

AFM has been instrumental in studying the motion of single molecules adsorbed onto a substrate, shedding light on the mechanisms behind preferential sliding directions. One striking example of friction anisotropy at the molecular level is observed when sliding a single para‐sexiphenyl (6P) molecule on an Ag(111) single‐crystal surface. 6P consists of six π‐conjugated phenyl rings arranged in a linear chain and is widely studied for its potential in optoelectronic applications.^208^ Despite having an identical contact area and interacting with the same underlying surface geometry, the lateral force required to move the molecule along its molecular axis is found to be about half of that required to move it sideways (Figure 8). The origin of this friction anisotropy lies in the molecular shape and the interfacial potential with the substrate. When 6P adsorbs onto Ag(111), molecule‐surface attractive interactions lower the potential energy beneath the molecule. As the molecule moves

- along its molecular axis (parallel direction): only the leading π‐ring encounters the potential barrier posed by the exposed Ag(111) surface.
- Sideways (perpendicular direction): all π‐rings must overcome this potential barrier simultaneously.

Although the number of surface atoms interacting with the molecule remains constant in both cases, the barrier to motion is significantly higher in the sideways direction, leading to a pronounced friction anisotropy.^209^

**FIGURE 9:**
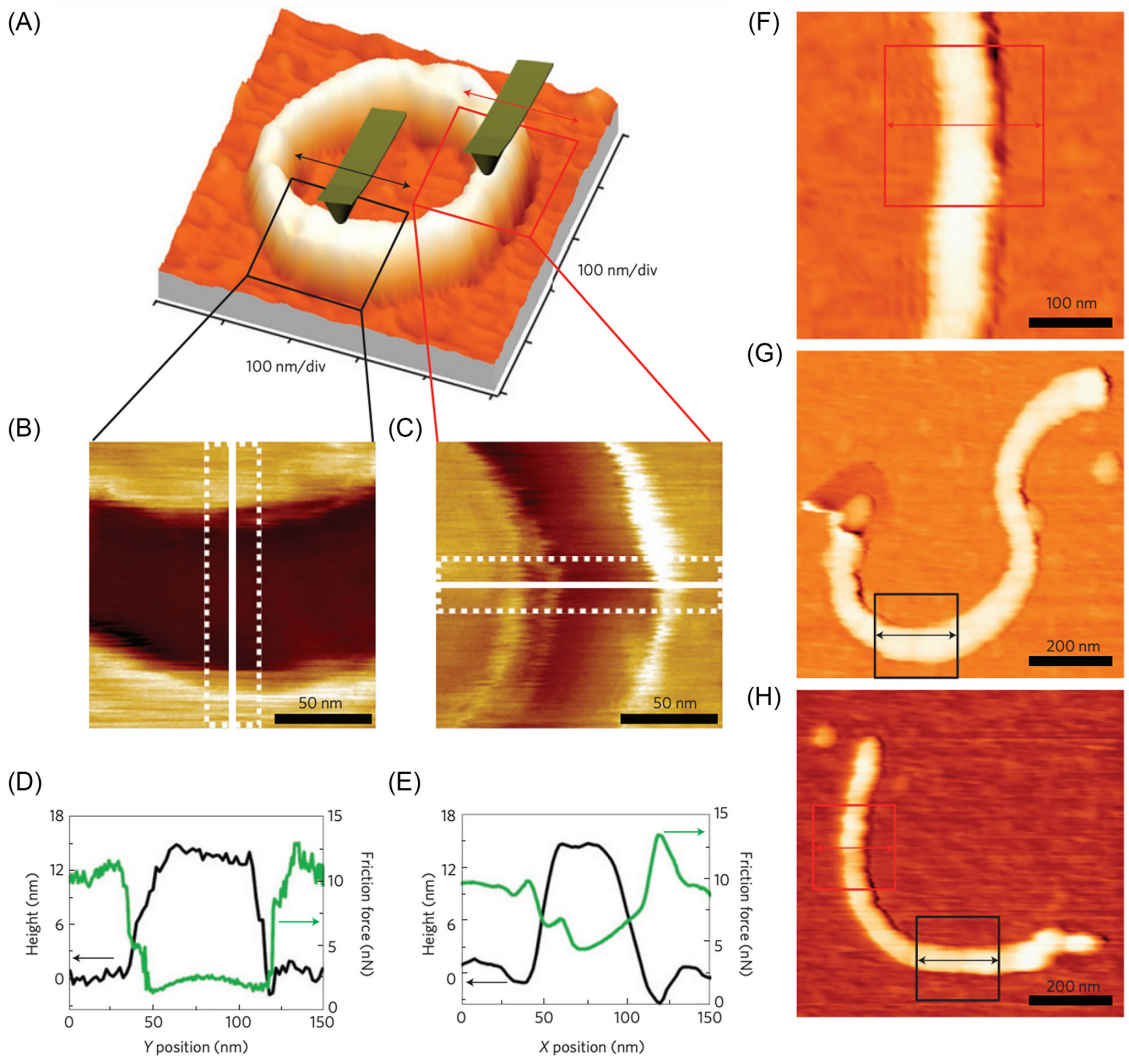
Friction measurements on CNTs. (A) Topography image of a ring‐shaped CNT of 7 nm radius. The fast‐scanning direction of the AFM tip is indicated by an arrow (*X* direction). (B, C) Friction images of the highlighted longitudinal (B) and transverse (C) sections of the nanotube. (D, E) Topography and friction force profile across the CNT. The topography profile (black solid line) corresponds to the white solid lines in (B, C), while the friction force profile (green solid line) represents the average force profile within the area delimited by the dotted line in (B, C). The friction force profile in (D) is measured along the Y direction, whereas in (E) is measured along the *X* direction. (F–H) Topography images of three additional CNTs with radius 11, 9, and 6 nm, respectively, where friction measurements were conducted within the areas outlined by solid lines. Adapted from Ref. (57).

The complexity of friction anisotropy increases when a larger number of solid adsorbates are deposited onto a substrate, as molecular interactions come into play alongside the surface potential with both the substrate and the sliding probe. A valuable model system for studying the impact of collective effects on friction anisotropy is carbon nanotubes (CNTs). Beyond their role as a model system, CNTs are of significant technological relevance. They have garnered considerable interest as solid lubricant additives in nanomechanical systems,^210^, ^211^, ^212^ and as potential components in nanoelectrical systems.^213^ Experiments involving the sliding of a silicon probe over multiwall CNTs deposited on a flat silicon substrate reveal a higher COF in the transverse direction compared to the parallel direction. This effect arises because transverse sliding induces a soft ‘hindered rolling’ of the nanotube, serving as an additional dissipation channel. In contrast, when the probe slides parallel to the CNT axis, this dissipation mechanism is either absent or significantly reduced, particularly for chiral CNTs (Figure 9).^57^ The tendency of CNTs to roll is further constrained by substrate adhesion, and this effect becomes more pronounced as the nanotube radius increases. A similar friction anisotropy trend has been observed for multiwalled boron nitride nanotubes (BN‐NTs). These BN‐NTs, synthesised via CVD and deposited onto a silicon substrate, were studied using an AFM probe sliding along their principal axis (longitudinal sliding) and perpendicular to it (transverse sliding). As with CNTs, transverse sliding produces greater friction coefficients, attributed to tube deformations during tip movement. Additionally, the contact area plays a crucial role in friction anisotropy: larger contact areas enhance surface adhesion, bringing the longitudinal friction coefficient closer to the expected value in the absence of transverse deformations.^214^

**FIGURE 10:**
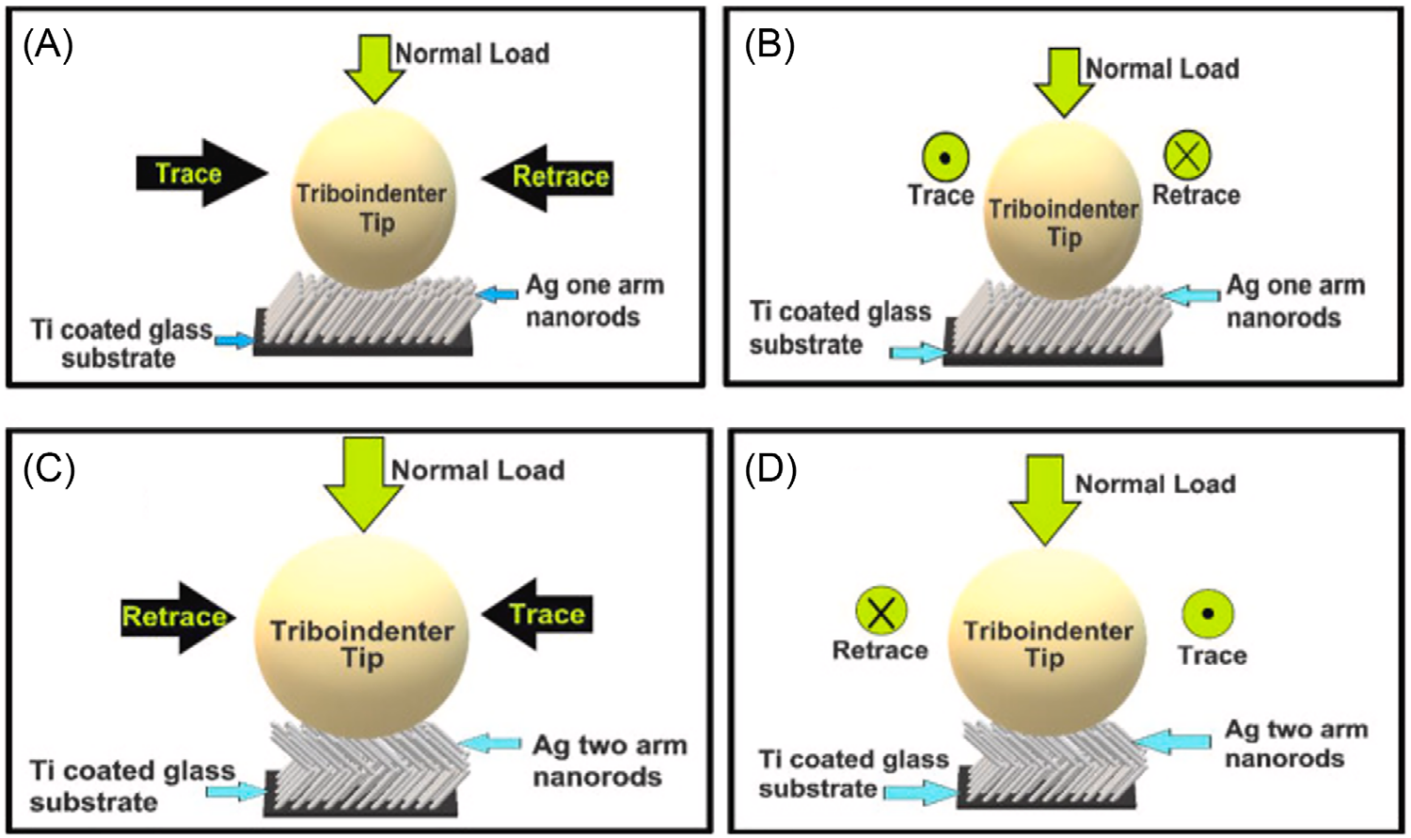
Schematic illustration of the experiments performed in Ref. (215). The AFM tip slides over one‐arm (A, B) and two‐arm (C, D) silver nanorod arrays, which are deposited on a Ti‐coated glass substrate. Panels (A) and (C) depict the motion of one‐arm and two‐arm nanostructures, respectively, along the columnar tilt direction during the trace (along the tilt) and retrace (against the tilt) scans. Panels (B) and (D) illustrate the motion of one‐arm and two‐arm nanostructures transversely to the columnar tilt, with the tip moving outwards from the plane of the page during the trace scan and inwards towards the plane of the page during the retrace scan. Adapted from Ref. (215).

Deformation and contact area have been identified as key factors influencing the observed friction response of metal nanorod arrays deposited on flat surfaces. In the case of single‐arm silver nanorods (Figure 10A and B), friction is found to be lower when the motion occurs along the tilt direction of the columns, rather than transversely. This is due to the ease with which the rods can compress along their tilt axis. However, for two‐arm nanocolumns (Figure 10C and D), friction anisotropy follows an opposite trend, with higher friction observed along the tilt direction. This suggests that smooth deformation is hindered by the energy cost associated with one arm bending over the other.^215^ Additionally, friction was found to vary depending on the scanning direction. Specifically, for one‐arm nanorods, friction was generally higher during forward scanning (trace) compared to backward sliding (retrace).^215^ In this system, friction is not only anisotropic (depending on the angle of the tip's motion relative to the domain orientation), but also asymmetric, that is, changing between the trace and retrace movements of the probe along the same scan line.^60^, ^69^

Studies involving nanorods made from silver and other metals (such as molybdenum and titanium) have identified an opposite trend: compressive deformation along the longitudinal direction and significant deflection against the column tilt result in higher friction resistance during reverse scratching compared to forward scratching. This is attributed to the more compliant bending deformation associated with the column tilt in forward motion.^216^, ^217^, ^218^ The different friction asymmetry observed can likely be explained by variations in the scanning tip geometry and the resulting contact area.^215^ In Refs. (216, 217, 218), relatively sharper or conical probes with radii of curvature between 50 nm and 100 µm were used, whereas Ref. (215) employed a larger, spherical probe with a radius of curvature of 400 µm. When a sharp conical probe moves along the tilt direction, it can easily penetrate in between the nanorod arrays instead of sliding over the patterned surface. However, when it moves against the tilt direction, apart from penetrating in the arrays, it causes deflection of the nanorods against the tilt which in turn generates more resistance to the motion of the probe. The scenario is reversed in the case of a spherical probe, significantly larger in size compared to columnar diameter; in this case, when moving along the tilt direction, the probe compresses the structures collectively instead of penetrating between them; a larger number of nanorods thus come in contact with the tip, increasing the total contact area and hence the friction force.^215^ The importance of contact area is further highlighted by the observation that, even with sharp probes, the relative difference in COF values when sliding across and along the tilt directions tends to increase with the applied normal load.^217^

**FIGURE 11:**
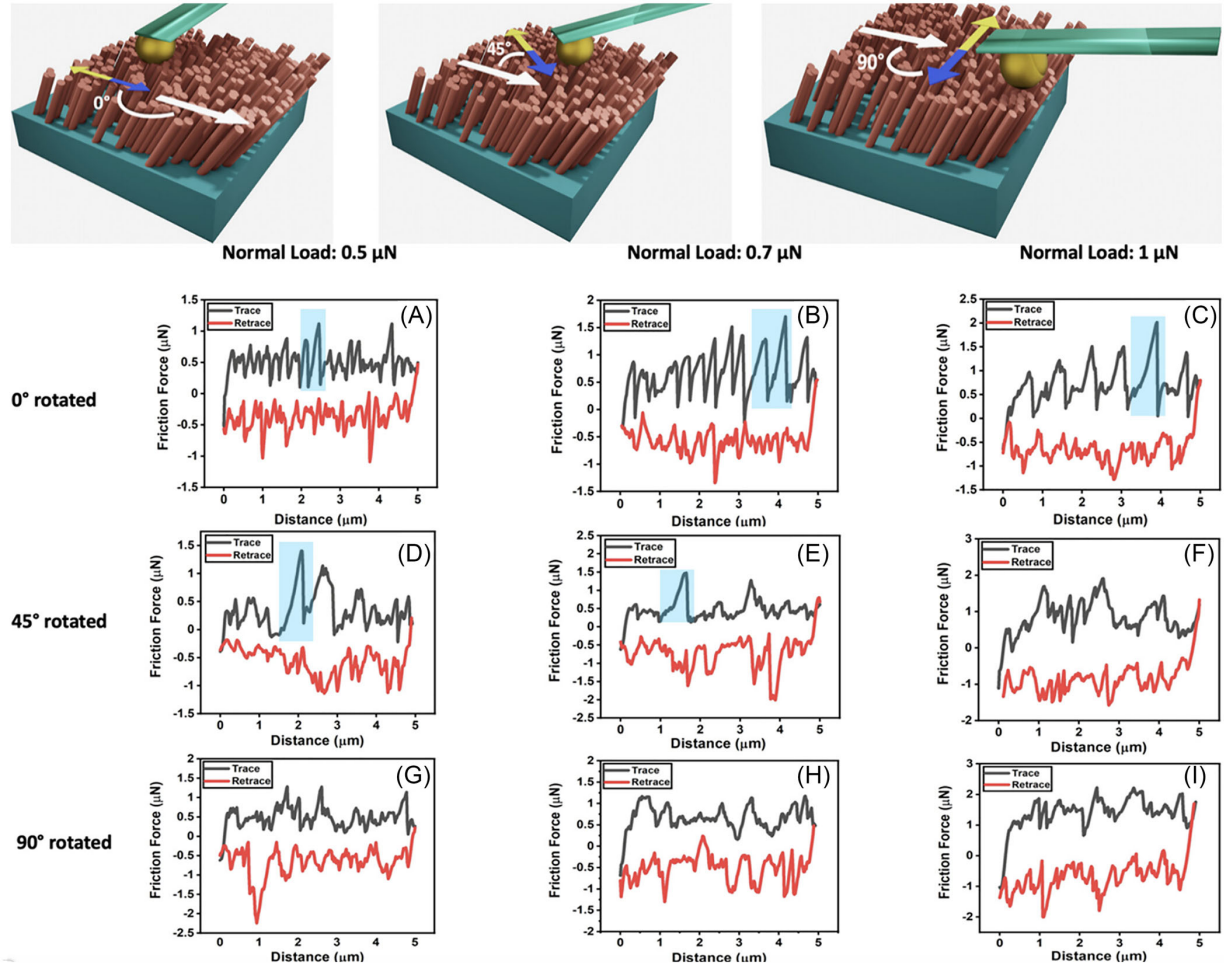
Direction‐dependent stick‐slip behaviour on tilted titanium nanorods. The top plot illustrates a schematic of the experiments conducted in Ref. (219) using a colloidal alumina probe. The white arrows indicate the direction of tilt of the titanium nanorods, while the double‐sided arrows represent the movement of the AFM cantilever. The yellow and blue sections of the double‐sided arrows correspond to the trace and retrace motion of the cantilever, respectively. The positions of the substrate are shown at 0°, 45°, and 90° rotations relative to the sliding direction. The bottom subplots (A–I) present the friction loops obtained after measuring the directional friction at different normal loads. Stick–slip behaviour is observed at the 0° rotated position, while it is suppressed at the 45° rotated position and almost eliminated at the 90° rotated position. These observed frictional characteristics are attributed to the geometry of the spherical probe pressing against the tilted rods, which causes lateral displacement. This interaction explains why the stick–slip behaviour varies with the rotation of the substrate. Adapted from Ref. (219).

Experiments with titanium nanorods and nanocolloidal probes have also revealed another contributing factor to friction anisotropy: the direction of sliding can influence the stick–slip behaviour. The frequency of stick–slip events decreases as the sliding direction moves from alignment with the tilt direction to perpendicular to it. The spherical probe presses against the tilted rods and laterally displaces them, which explains the variation in stick–slip behaviour with the rotation of the substrate. When aligned with the nanorods’ tilt angle, the correlation length matches the maximum horizontal deflection of the rods when pushed against the tilt direction (Figure 11).^219^

### 3.2 Polymers, lipids, and SAMs

**FIGURE 12:**
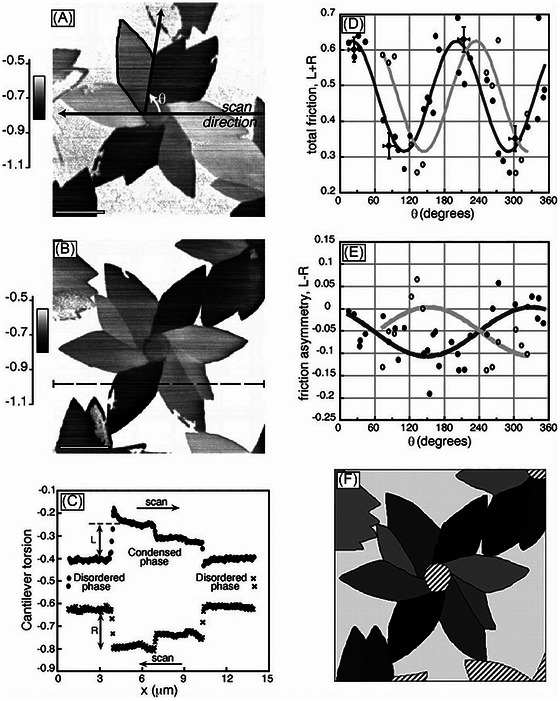
LFM characterisation of a thiolipid monolayer on a mica surface. (A) LFM image showing a flower‐shaped condensed domain against the disordered phase. The internal structure of the domain is revealed by high friction contrast between the different petals. The angle *θ* between the lower right petal boundary and the scan direction defines the orientation of the outlined petal. High lateral forces correspond to dark grey shades. Scale bar, 4 µm; scan velocity, 42 µm/s. (B) As for (A), after counterclockwise rotation of the sample by 70°. The dashed line represents the scan line of the friction loop in (C). (C) A typical friction loop (arbitrary torsion units). The upper curve represents a scan from left to right, the lower curve from right to left. The friction force amplitudes *L* and *R* on the condensed domain are defined relative to the disordered phase and are typically 1 to 2 nN. For measurements of the friction forces on the individual petals, 15 adjacent friction loops are averaged. Values of *L* and *R* yield the total friction *L* + *R* and the friction asymmetry *L* − *R*. The overshoots seen in some regions of the curve are an artefact caused by the height difference of 1.5 nm between the domains and the matrix. (D) Total friction (*L* + *R*) versus the orientation of the major petals (filled circles) and minor petals (open circles) with respect to the scan direction as defined in (A). The fit for the major petals (dark line) is based on a sinusoidal curve with a periodicity of 180°; all other parameters were unconstrained. In the fit for the minor petals, the only free parameter is the phase angle. All other parameters are taken directly from the major petal fit. The phase shift between the curves reflects the definition of *θ*. Relative to the major petals, the subdomain boundaries of the minor petals are displaced by 30°. (E) Friction asymmetry (*L* − *R*) versus the orientation of the major and minor petals, as in (D). Here, the difference in phase for the major and minor subdomains is between 150° and 210°. (F) A simulated friction force image. The phase angle of (*L* + *R*) is chosen to give the first maximum at 30°; the phase angle of (*L* −*R*) is chosen to give zero asymmetry at 30° and 210°. The ratio of the amplitudes of total friction and friction asymmetry is 10:1. These parameters lead to a good agreement between the simulated images and experimental results, as seen by comparing with the corresponding experimental image in (B). Opposing pairs of petals, oriented roughly perpendicularly to the horizontal scan direction, exhibit similar grey levels, while pairs oriented approximately horizontally show a noticeable difference in grey level. Adapted from Ref. (60).

Friction anisotropy is also observed when deposited oblique columns are made from polymers rather than metals. In the case of parylene polymers, friction is higher when sliding perpendicular to the column tilt axis, with minimal friction asymmetry. However, sliding parallel to the tilt axis results in both friction asymmetry and depth hysteresis, characterised by larger contact depths and higher COF along the column tilt direction.^220^ This behaviour is similar to that observed in metal‐based nanorods,^215^, ^216^, ^217^, ^218^ with the use of a relatively blunt probe potentially accounting for the findings.^220^ A similar trend has been reported when probing a thiolipid monolayer deposited on muscovite mica,^60^, ^221^ where the alkyl chains are tilted approximately 15° from the surface normal.^60^ Friction forces in this case also exhibit anisotropy (asymmetry) with higher friction when sliding perpendicular to (along) the tilt axis (Figure 12).^60^ In these experiments with thiolipids, a relatively sharp probe is, however, used. Therefore, the typical compression/penetration mechanism cannot explain the results, as it would predict high friction against the tilt direction for sharp probes and high friction along the tilt direction for blunt probes or those with larger radii of curvature. The apparent contradiction may be resolved with the following hypothesis: the softer nature of the alkyl chains, compared to polymeric or metal nanorods, could lead to a larger effective contact area between the lipids and the sharp probe. In this case, the system would behave similarly to stiffer nanorods probed by larger, blunt tips,^4^ supported by analogous findings in similar systems, such as SAMs of either mono‐alkyl glycerol molecules^222^, ^223^ or liquid crystals.^224^

It is important to highlight that, as suggested by these studies,^60^, ^222^ the viscoelastic response of the adsorbed polymers^41^, ^225^, ^226^ adds a layer of complexity to the problem of friction anisotropy, underscoring the importance of experimental studies to validate and identify the limitations of any hypothesised general mechanisms. This is confirmed by experiments on a polydiacetylene monolayer,^61^ which emphasise the importance of both the viscoelastic properties of the adsorbed molecules and their molecular packing.^52^, ^227^, ^228^, ^229^, ^230^ In the study from Ref. (61), the monolayer forms domains with linearly oriented conjugated backbones, surrounded by pendant hydrocarbon side chains above and below the backbones. These backbones impose an anisotropic packing of the side chains, leading to friction anisotropy, with maximum friction observed when the sliding direction is perpendicular to the backbone.^61^ Similarly, in polyethylene single crystals, friction anisotropy is thought to arise from the fact that different folding directions of the polymer chains impart anisotropic elastic properties to the material.^231^

Additionally, the physical‐chemical properties of certain lipid bilayers and their interactions with the substrate can promote a solid‐like alignment of the adsorbed molecules into highly ordered structures that exhibit nearly crystalline characteristics. A representative example is 5‐(4'‐N, N‐dihexadecylamino) benzylidene barbituric acid, transferred via the Langmuir‐Blodgett (LB) technique onto an oxidised silicon (100) substrate. In this case, the ordering of the lipid moieties into a bilayer results in a nearly crystalline structure under ambient conditions. The direction‐dependent corrugation of this structure explains the observed friction anisotropy: the anisotropic intermolecular spacings in the lipid bilayer alter the interaction potential between the AFM probe and the bilayer depending on the sliding direction, corresponding to different molecular alignments.^232^

The principle that ordering at the interface governs friction anisotropy is general and extends beyond molecular films. For example, the interaction of an adsorbed layer with a substrate can also induce anisotropic friction in inorganic systems. This is exemplified by Ref. (233), where an inorganic KBr thin film is deposited on a Cu(100) single crystal. In this case, friction force reveals two patterns with cubic symmetry: one corresponding to the periodicity of the KBr lattice and the other to its superstructure.^233^

**FIGURE 13:**
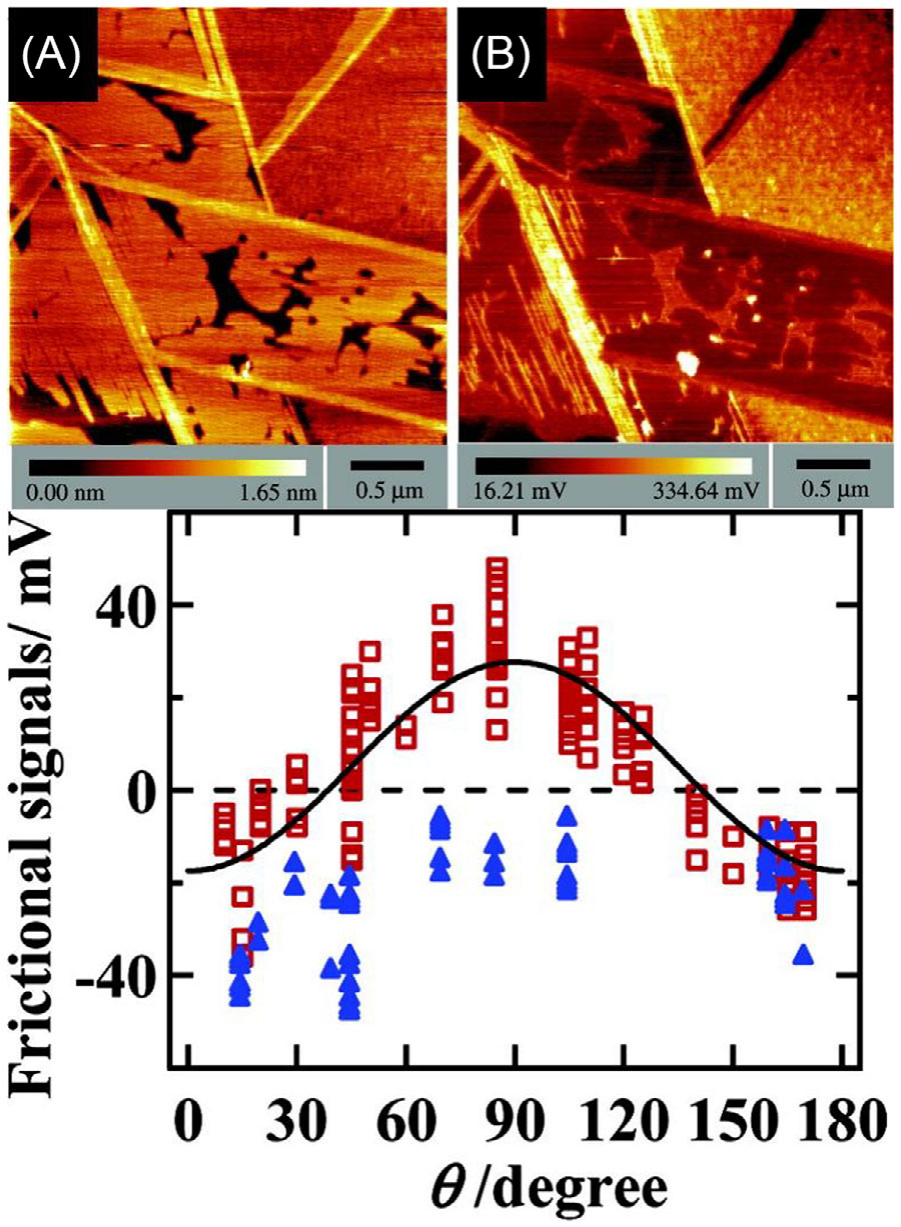
Friction anisotropy in organogelator films on mica. (A) Topographic and (B) lateral friction force microscopy images of a model organogelator film deposited on mica substrate by LB method. The bottom plot illustrates the frictional signal as a function of the angle between the scanning direction and the oriented directions of the long axis of the fibrous structure (red squares) or the uniaxial molecular assembly (blue triangles). Friction anisotropy is observed on the fibrous structure, with the maximum friction occurring in the perpendicular direction due to the molecular tilt orientation relative to the sliding probe (see also Figures 10 and 11). Adapted from Ref. (234).

The fundamental impact of molecular packing on friction has been shown also in the case of other LB films, SAMs, polymeric layers and thin films, and liquid crystals.^54^, ^200^, ^221^, ^230^, ^234^, ^235^, ^236^, ^237^, ^238^, ^239^, ^240^, ^241^, ^242^, ^243^, ^244^, ^245^, ^246^, ^247^, ^248^, ^249^, ^250^, ^251^, ^252^, ^253^ For example, low molecular weight organogelators – a class of polymers with a liquid phase within a 3D network structure – form quasi‐1D fibres that are entangled with one another.^234^, ^254^ These materials have broad applications, from solar cells^255^ to marine oil spill remediation.^256^ When organogelators are deposited onto muscovite mica, uniform domains and fibrous structures form. In domains where the organogelator molecules are disordered, no friction anisotropy is observed. However, when the molecules form an epitaxial crystalline, fibrous structure, the friction is minimised along the scanning direction parallel to the long axis of the fibres and maximised along the perpendicular direction (Figure 13).^234^ In this case, friction arises from the fibre tilting at the surface, similar to what is observed for lipid^242^ and thiolipid molecules,^60^ where friction is greater when sliding perpendicular to the tilt direction compared to the parallel direction. This trend has been further confirmed in systems such as polyethylene and poly(tetrafluoroethylene) polymers, where the friction response perpendicular to the polymer chains is significantly higher than the friction measured along the chains.^249^ In addition to influencing the preferential elasticity and bending stiffness, the molecular ordering of the moieties can also impact friction anisotropy by exposing different functional groups, which in turn modify the interactions with the scanning probe.^252^

The impact of polymeric molecular ordering on friction anisotropy can also be attributed to the different state of the polymer. The coexistence of fluid and solid domains within the samples corresponds to different frictional forces. Intuitively, solid domains are expected to generate higher dissipation, whereas the fluid phase should act as an effective lubricant, promoting smoother sliding of the probe.^27^, ^257^ Interestingly, while some studies on thiolipids have confirmed this expected trend,^221^ others have reported opposite behaviour, with higher friction observed when scanning along directions corresponding to thiolipids fluid phase.^258^ While factors such as thermal effects, substrate condition, preparation methods and tip‐convolution effects cannot be entirely ruled out,^239^ the apparent discrepancy may stem from the fact that, despite their structural similarity, the thiolipids used in these studies were different, leading to variations in their interfacial potentials. In Ref. (221), greater friction on the solid domains may be due to solid–solid interlocking effects. Conversely in Ref. (258), the solid–solid interfacial barrier is smaller than the energy barrier associated with the viscosity forces experienced by the sliding probe, resulting in greater pull off force in the fluid phase. This underscores the critical role of molecular packing in friction anisotropy, with the specific chemical‐physical characteristics of the moieties influencing the molecular ordering and their response to applied shear forces.

**FIGURE 14:**
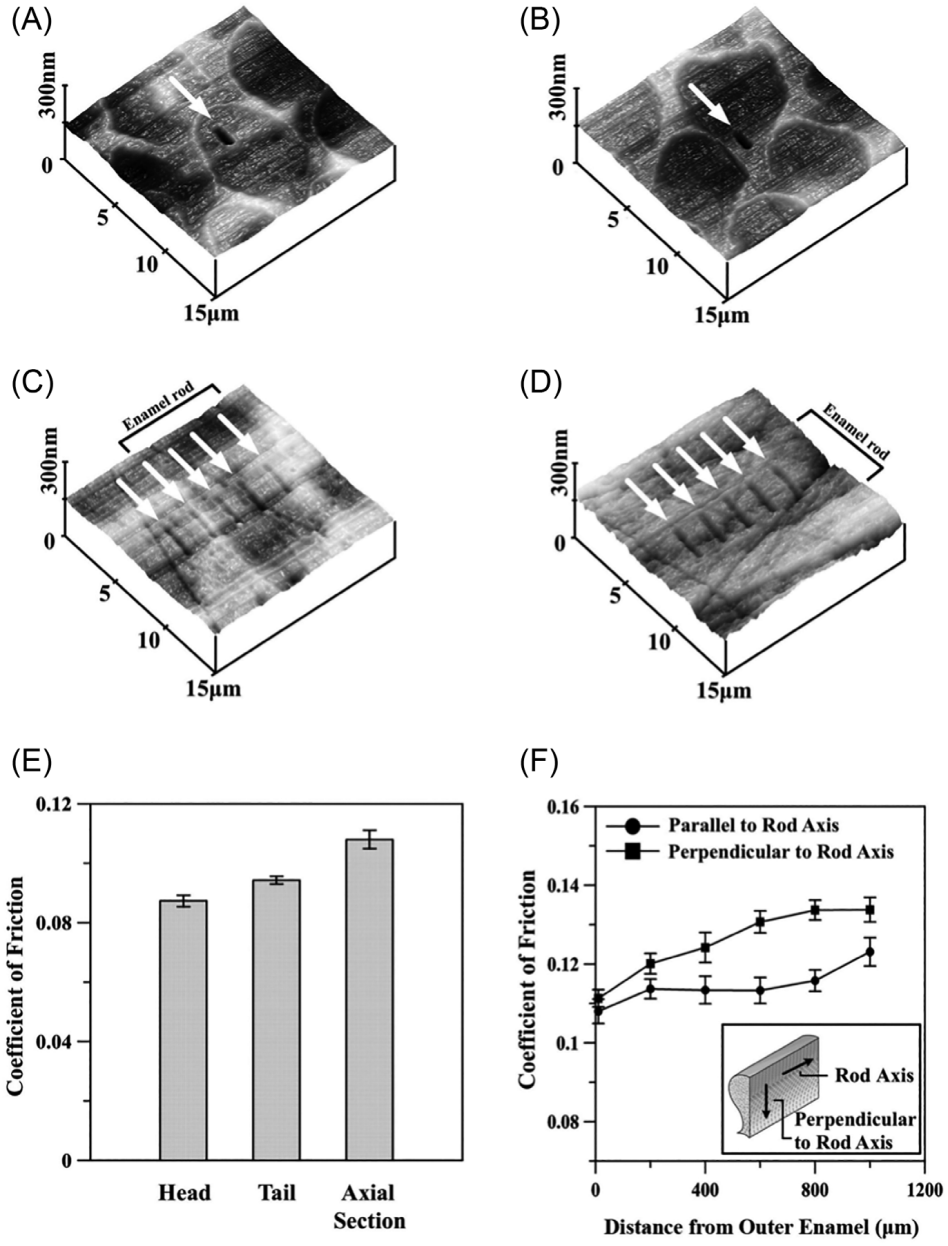
Friction anisotropy of enamel rods as measured by nanoscratch testing and friction coefficient analysis. AFM images showing: (A) a representative nanoscratch performed in the head region of the enamel rod, (B) a scratch mark in the tail region of the rod, (C) a nanoscratch marked along the longitudinal axis of the enamel rod in the axial section, and (D) a nanoscratch performed in the axial section with a direction perpendicular to the longitudinal axis. (E) Friction coefficients measured from different topological regions of the enamel rods. (F) Friction coefficient variation along the longitudinal axis of the enamel rod, from the outer enamel surface to the dentinal–enamel junction. Adapted from Ref. (259).

Considering biological or bio‐inspired polymeric surfaces, the ordered directional texture plays a key role in friction anisotropy and asymmetry at the nano‐ and micro‐scale, from human teeth enamel rods^259^ and snake skin micro‐hair^260^, ^261^ to synthetic surfaces mimicking biological systems.^262^ The hydroxyapatite fibre like crystals arrangement of enamel rods results in higher friction when sliding perpendicular to the rod axis in comparison to sliding parallel to it (Figure 14).^259^

The micro‐hair surface structure of snakeskin, organised into ordered arrays of microfibrils, provides a mechanism for flexible rearrangement of sliding orientation. On one hand, the ‘double‐ridge’ design of the microfibrils significantly reduces adhesive forces at contact areas, creating ideal conditions for forward motion with minimal friction and adhesive forces.^260^ On the other hand, the asymmetric ‘geometrical’ design of the microfibril ends effectively acts as a stopper for backward motion, with friction anisotropy increasing as the height of the microfibrils rises.^260^, ^261^ These findings have inspired the development of bio‐inspired surfaces, such as polymer‐based microfibrils. The patterned microfibrils, with their nanosteps, lead to tuneable friction anisotropy, which increases with the nanostep height.^261^, ^263^ Similar dynamics are observed in other biological systems, where the frictional response is influenced by the structural characteristics and surface patterning. For example, the toe pads of tree frogs,^264^ gecko spatulae^265^ and the cockroach tarsal euplantulae^266^ all exhibit surface patterns that modulate friction. Let's focus on the cockroach tarsal euplantulae as a representative system. Their surface is covered with asymmetrical ridges approximately 200 nm high, with steeper slopes facing distally and shallower slopes proximally. This arrangement facilitates interlocking with rough substrates when pushed distally, while enabling easier slipping when pulled proximally.^266^

**FIGURE 15:**
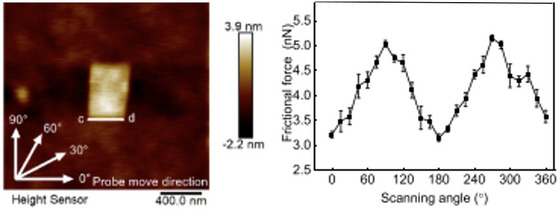
Friction anisotropy of a violet phosphorene nanoflake adsorbed on silicon. On the left, an AFM topographical image shows a violet phosphorene nanoflake adsorbed onto a silicon substrate, with white arrows indicating different sliding directions of the AFM probe relative to the cleavage edge. On the right, the friction anisotropy of the system is depicted. Adapted from Ref. (267).

Building on the role of ridges in cockroach tarsal euplantulae,^266^ it is important to note that friction, despite its complexity, shares a fundamental principle across various surfaces, from biological systems to 2D materials: the breaking of in‐plane symmetry at the interface. While in biological and bio‐inspired systems this symmetry breaking is governed by surface morphology (e.g. ridges or fibrils), in 2D materials it can emerge from the anisotropic electronic and phononic structure of the lattice. For instance, in violet phosphorene nanoflakes, two sets of parallel sub‐nanorods are arranged in distinct planes with 180° periodicity (Figure 15). The surface structure of violet phosphorene shows the least resistance to a probe when it moves along the green sub‐nanorod direction (at scanning angles of 0° or 180°). In contrast, the greatest resistance occurs when the probe moves across the green sub‐nanorod direction (at scanning angles of 90° or 270°). This variation in friction response is linked to the distribution and deformation anisotropy of electron and phonon states on the surface of violet phosphorene (Figure 15).^267^

A further example of how both hard and soft matter systems share similar key mechanisms behind friction anisotropy can be seen by comparing cellulose nanocrystals (CNCs)^268^, ^269^ and KBr thin films on a Cu(100) single crystal.^233^ The substrate lattice plays a critical role in modulating the anisotropic ordering of the molecules, and thus their direction‐dependent frictional response. CNCs, which can be extracted from bacterial cellulose pellicles as raw material, naturally self‐order into rod‐like structures. This self‐organisation leads to the formation of liquid crystals, with different alignments of the liquid crystal domains corresponding to sliding directions that exhibit low and high friction.^269^ Interestingly, the friction anisotropy of CNCs can be influenced by interactions with the substrate's corrugation, allowing for further modulation.^268^ Similarly, the epitaxial effects of Cu(100) single crystals on KBr thin films^233^ highlight a key principle behind friction anisotropy across different material classes: the interactions between the deposited molecules and the substrate result in anisotropic ordering. This mechanism also plays a fundamental role in friction anisotropy in liquid adsorbates, as discussed in the following section.

### 3.3 Fluid adsorbates

Fluid‐based lubricants are ubiquitous, from synovial joints^270^, ^271^ to car and machinery engines.^4^ Given their technological importance, understanding the molecular mechanisms behind friction anisotropy at fluid‐lubricated solid interfaces is essential. Friction anisotropy can be advantageous in some scenarios, such as in nanorobotics,^4^, ^32^ where modulating friction between high and low states is crucial to the device's functioning. However, in many applications, such as car engines, inhomogeneous lubrication can hinder the smooth motion of engine parts, negatively impacting energy efficiency.^272^

AFM studies have significantly advanced our understanding of the mechanisms behind friction anisotropy in fluid‐lubricated interfaces.^26^, ^27^, ^31^, ^32^, ^34^, ^35^, ^45^, ^50^, ^273^, ^274^, ^275^, ^276^

Like polymers and self‐assembled monolayers, fluid adsorbates exhibit friction anisotropy primarily due to their anisotropic spatial arrangement. While molecular ordering and its influence on friction anisotropy represent general principles, several factors influence this ordering. These factors can be grouped into three main categories: (1) the physical‐chemical properties of the fluid lubricants, (2) the interactions between the fluid and the substrate, and (3) the experimental conditions (e.g. temperature and humidity).

**FIGURE 16:**
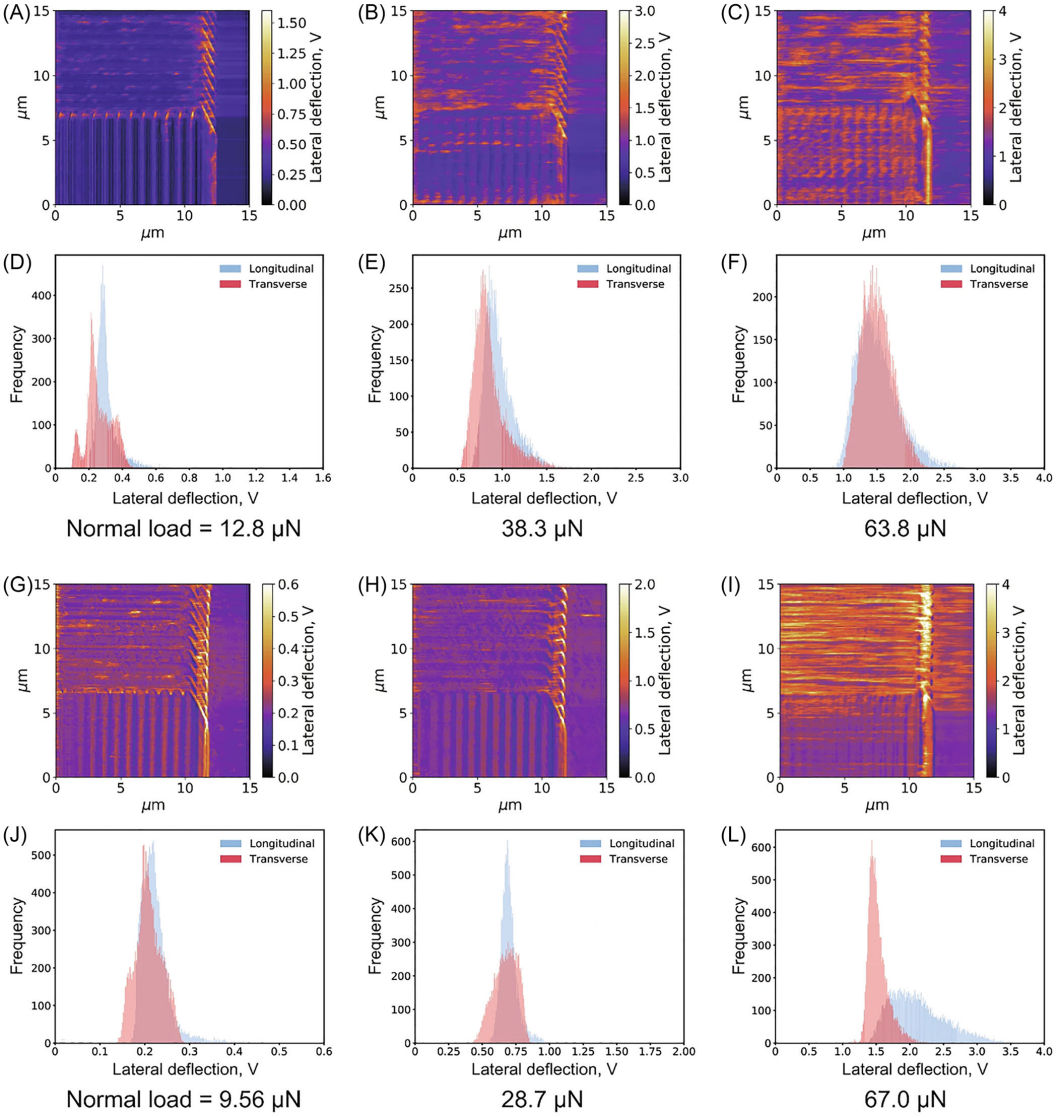
Boundary‐lubricated friction in *n*‐hexadecane (A–F) and stearic acid (G–L) between an AFM probe and a Si(100) surface micropatterned by photolithography. The friction is assessed in the boundary lubrication regime on the patterns using in‐liquid LFM, with measurements taken for both transverse and longitudinal ridges, relative to the sliding direction. In panels (A–C) and (G–I), LFM images are shown with the upper‐left and lower‐left parts corresponding to the longitudinal and transverse ridge regions, respectively. Panels (D and F) and (J–L) display LFM signal histograms extracted from the longitudinal and transverse ridge regions in (A–C, G–I). For *n*‐hexadecane (A–F), no noticeable variation in friction is observed between the longitudinal and transverse ridge regions across all applied loads. In contrast, the results for stearic acid (G–L) reveal differences in frictional force depending on the ridge direction at an applied load of 67.0 µN, while no clear difference is observed at lower loads. Interestingly, the highest friction is found on the longitudinal ridges and grooves. On patterns where fluid cannot flow along the grooves, frictional forces are equivalent for both transverse and longitudinal ridges and grooves. The elevated friction observed on the longitudinal ridges is attributed to the fluid flowing out along the grooves. This suggests that the fluid's behaviour around the sub‐micrometre ridges and grooves plays a crucial role in modulating the friction‐reducing effects of stearic acid in the boundary lubrication regime. Adapted from Ref. (277).

To illustrate the first variable, consider a study where a Si(100) surface was micropatterned using photolithographic techniques and probed with AFM tips with a blunted area of a few micrometres.^277^ Two fluids were tested: *n*‐hexadecane and stearic acid. The former showed no direction‐dependent friction response (Figure 16A–F), while the latter exhibited significant friction anisotropy (Figure 16G–L). The difference in the melting point and adsorption properties of *n*‐hexadecane^26^, ^41^ and stearic acid^278^ may account for their different impact on friction anisotropy. Stearic acid has a higher melting point in comparison to hexadecane and may remain in a more fluid‐like state at nanoconfinement. With its relatively low melting point, *n*‐hexadecane may form a stably adsorbed layer ensuring effective lubrication along all the probed sliding directions. Interestingly, friction anisotropy in stearic acid was characterised by higher friction when sliding along the micropatterned ridges, rather than perpendicular to them (Figure 16I and L). This result may seem counterintuitive, as simple geometric effects from substrate defects alone are not enough to explain the system's behaviour. The reduced lubrication along the defects can be explained by the shear force applied by the probe, which acts over a longer period, effectively squeezing the stearic acid fluid out of the nanogaps.

**FIGURE 17:**
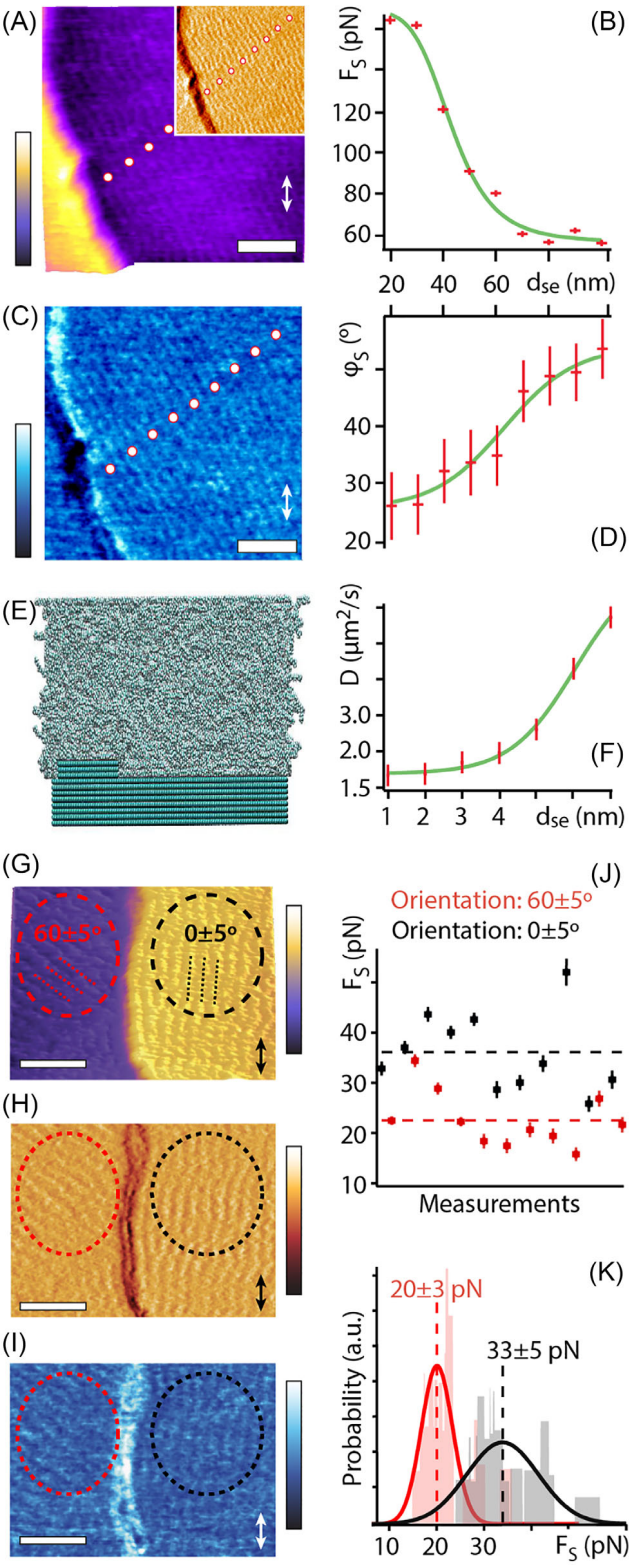
Shear behaviour of squalane molecules near an HOPG step edge at 298 K. The row‐like arrangement of squalane molecules parallel to the edge is evident over the whole AFM images (A and C). The inset in panel (A) highlights variations in the scanning amplitude where the contrast over the rows is more distinct. Shear force spectroscopy measurements taken at set distances *d*_SE_ from the step reveal a decrease in lubricated friction force (shear force *F*_S_) (B) and increase in the shear phase *φ*_S_ (D) when moving away from the step. *F*_S_ and *φ*_S_ are taken at an applied lateral force *F*_L_ ∼ 30 nN. The shear direction is illustrated by the white double‐headed arrows (A and C). The shear force *F*_S_ is an absolute measurement of the average lubricated friction experienced by the tip, and the shear phase *φ*_S_ quantifies the viscoelastic properties of the confined lubricant (not to be confused with the imaging phase). A value of *φ*_S_ = 0° indicates a purely elastic behaviour of the sheared squalane layer, whereas 90° corresponds to a purely viscous behaviour. Consistently, molecular dynamics (MD) simulations (unit cell of 34.1 nm × 7.2 nm × 11.8 nm with ∼3000 squalane molecules) (E) show an increase in squalane diffusion constant, *D*, when moving away from the step edge at the interface (averaged within 1.2‐nm layer above the HOPG surface) (F). Quantitative comparison between AFM and MD measurements is difficult because of limited size of the simulation box. Scale bars, 25 nm. The colour bars in (A) and (C) represent height variations of 1.2 nm (0.6 nm for inset) and phase variations of 3.0°, respectively. (G–K) The impact of molecular ordering direction on the lubricated friction force. Panels (G–K) demonstrate the impact of molecular ordering orientation on lubricated friction force. High‐resolution topographic (G), amplitude (H), and phase (I) AFM images of the molecular arrangement of squalane at the interface with HOPG near a step. Domains with different row orientations are visible (dashed red and black circles). Shear force measurements show a clear sensitivity to row orientation with statistically higher *F*_S_ values when shearing parallel to the rows (J and K). Multiple measurements taken over the two regions (G–I) statistically confirm the friction dependence on the rows orientation (K). Scale bars, 25 nm. The imposed shear direction is indicated with a double‐headed arrow (G–I). The dashed lines in (J) and (K) represent mean force values. The colour bars represent total variations of 5.0‐nm height (G), 0.2 nm (H), and 10.0° (I). The shear forces in (J and K) are taken at an applied load of *F*_L_ of 12 nN. a.u., arbitrary units. Adapted from Ref. (27).

The inability of geometric effects alone to fully explain tribological phenomena in fluid‐lubricated systems brings us to the second set of variables influencing liquid ordering: the interactions between the liquid and the substrate. This is demonstrated in Ref. (27), where squalane is nanoconfined between a diamond‐like carbon AFM probe and a freshly cleaved HOPG surface (Figure 17A–K).

HOPG step edges promote the local molecular ordering of squalane molecules into row‐like nanodomains (Figure 17A and C). The surface defects effectively reduce the entropy of the squalane molecules, limiting the number of stable molecular configurations in their immediate vicinity. As a result, the system exhibits a more elastic‐like behaviour and higher friction along the molecular rows (Figure 17B and D), even when compared to scanning directly over the surface defect itself. In other words, the increase in lubricated friction near a step edge is not simply a geometric effect due to the tip interacting with a rougher area but rather an indirect consequence of the localised molecular ordering induced by the surface features of HOPG (Figure 17E and F). The molecular ordering, driven by interactions with the solid substrate, leads to friction anisotropy, with higher friction occurring when shearing parallel to the row‐like domains. When the AFM probe moves parallel to the rows, it must disrupt the coherent molecular structure along its entire path, whereas shearing at an angle allows the probe to ‘section’ the row‐like features more easily, thereby reducing the frictional force experienced (Figure 17G–K).^27^

It is insightful to compare fluid‐lubricated systems and SAMs in terms of the impact of molecular ordering and packing density on friction anisotropy, as investigated by AFM. Both systems involve the adsorption of molecules onto a substrate. However, in SAMs, molecules form a stable mono‐ or bilayer on the substrate over which the probe slides. In contrast, the behaviour of lubricant molecules is more complex. The nanoconfinement created by the sliding probe induces a local solid‐like organisation or layering^4^, ^50^ increasing the relaxation constant of the molecules.^275^ Consequently, the lubricant properties change in response to this structural alteration. While the lubricant molecules retain their fluid characteristics laterally away from the probe or higher up from the adsorbed molecular layers, their behaviour near the probe becomes more solid‐like. For fluid adsorbates, the solid‐like organisation of fluid molecules driven by entropy reduction leads to an increase in friction force, whereas the liquid phase favours smoother sliding of the probe over the substrate.^27^ In contrast, in SAMs, high friction is observed in less‐ordered regions with lower packing density, as tightly packed molecular lattices restrict molecular movement due to strong intermolecular interactions. This restriction suppresses energy dissipation mechanisms such as molecular tilting, rotations, and gauche defects, resulting in reduced friction.^238^, ^245^ The structural differences between fluid‐lubricated systems and SAMs explain the distinct effects of molecular packing on friction in the two systems.

The third important set of variables influencing liquid ordering belongs to a heterogeneous category, encompassing contributions from experimental conditions, such as temperature and humidity. Temperature, for example, has been shown to affect friction anisotropy, as highlighted in the aforementioned study^27^ on the lubricated friction of squalane near HOPG nanodefects. Increasing the temperature progressively disrupts the molecular ordering of squalane into row‐like domains, especially as the distance from the confining step edge increases. Humidity can also interfere with the molecular ordering of fluid adsorbates,^41^ potentially impacting friction anisotropy as well.

## 4 CONCLUSIONS

AFM has been instrumental in elucidating friction anisotropy across a broad range of material systems, including atomically flat crystals, thin films, polymers, and fluid lubricants adsorbed on topographically complex surfaces. Table 1 provides an overview of the main classes of interfaces and materials investigated using AFM, as reviewed in this work, together with the primary mechanisms responsible for the observed anisotropic/asymmetric frictional behaviour.

**TABLE 1:** Overview of the principal classes of interfaces and materials examined in this review, along with the dominant mechanisms responsible for the observed friction anisotropy and asymmetry. For a detailed discussion and a complete list of references, please refer to the main text.

Interface	Material type	Representative systems	Mechanisms	Refs
Bare atomically‐flat surfaces	Atomic and ionic crystals	Alkali halides Alkaline earth sulphates and carbonates	Periodic corrugations due to ionic orientation	64, 96, 97, 98, 99
		Diamond	Density of amorphous defects	79, 101
	Quasicrystals	Decagonal Al‐Ni‐Co	Periodic and aperiodic directions	65, 103, 104
	2D materials	HOPG Graphene Black phosphorous *h*‐BN MoS_2_, MoSe_2_ WS_2_	Zigzag and armchair axes; puckering effects; formation of ripples and wrinkles; airborne contaminants forming regular features	67, 80, 120, 125, 126, 127, 128, 129, 130, 131, 132, 133, 134, 135, 136, 137, 138, 139, 140, 141, 142, 143, 145, 146, 147, 148, 149, 150, 151, 157, 161, 162, 163, 164, 166, 167, 168, 169, 170, 186, 187, 188, 189, 190, 191
		2D homo‐ and heterostructures	Lattice mismatch; chemical bonding	94, 173, 174, 175, 176, 177, 178, 179, 180, 181
	Organic molecular crystals and organic salts	L‐alanine, β‐alanine Potassium hydrogen phthalate	Grooves due to oriented molecular moieties	192, 193, 194
		Triglycine sulphate	Ferroelectric surface potential	204
Presence of adsorbates	Solid adsorbates	6P on Ag(111)	Molecule‐surface potential energy barrier	209
		CNTs and BN‐NTs on Si substrate	Hindered rolling due to geometry and deformation	57, 214
		Metal nanorods on flat surfaces	Contact area variation induced by deformation	215, 216, 217, 218, 219
	Polymers, lipids, SAMs and organic molecular films	Lipid bilayers and thiolipid monolayers Polymer films: parylene, polyethylene and poly(tetrafluoroethylene) Liquid crystals Organogelators on mica	Column tilt axis and viscoelastic properties due to anisotropic packing of side chains	52, 54, 60, 61, 200, 220, 221, 225, 226, 227, 228, 229, 230, 231, 232, 234, 235, 236, 237, 238, 239, 240, 241, 242, 243, 244, 245, 246, 247, 248, 249, 250, 251, 252, 253, 258
		Bio and bio‐inspired interfaces e.g. human teeth enamel rods, snakeskin micro‐hair, polymer‐based microfibrils	Flexible rearrangement and contact area of crystals and microfibrils	259, 260, 261, 262
	Fluid adsorbates	n‐hexadecane and stearic acid on micropatterned Si(100)	Different adsorption due to chemical properties, i.e. melting points	277
		Nanoconfined squalane	Entropy driven molecular ordering	27

Beyond elucidating the fundamental principles of friction anisotropy and asymmetry, AFM has been instrumental in testing and clarifying theoretical models, from commensurability and lattice mismatch to strain‐induced nanoscale defects. AFM has also highlighted the importance of integrating these principles with the specific chemical and physical properties of the interacting surfaces. Factors such as tip‐sample contact quality, entropy‐driven organisation of liquid lubricants around nanodefects and the preferential adsorption of airborne contaminants along given surface directions can all give rise to unexpected friction anisotropy and asymmetry.

Overall, AFM studies have demonstrated that, at the atomic, nanoscale and microscale level, friction anisotropy and asymmetry emerge from a complex interplay between periodic corrugation of the solid surfaces, deformation due to the surface defects, and the adsorption of lubricants and contaminants. Future research is encouraged to further bridge the current gap between atomic/nanoscale mechanisms and macroscale tribological behaviour, ultimately advancing our understanding of natural systems and optimising tribological solutions for enhanced energy efficiency.
